# Supplementary material for: Evaluating NHS Stop Smoking Service engagement in community pharmacies using simulated smokers: fidelity assessment of a theory-based intervention
Source: BMJ Open. 2019 May 19;9(5):e026841. doi: 10.1136/bmjopen-2018-026841 (PMC6530322; doi:10.1136/bmjopen-2018-026841)
Supplement: Author's manuscript [file bmjopen-2018-026841.draft_revisions.pdf]

BMJ Open

BMJ Open is committed to open peer review. As part of this commitment we make the peer review history of every article we publish publicly available.

When an article is published we post the peer reviewers' comments and the authors' responses online. We also post the versions of the paper that were used during peer review. These are the versions that the peer review comments apply to.

The versions of the paper that follow are the versions that were submitted during the peer review process. They are not the versions of record or the final published versions. They should not be cited or distributed as the published version of this manuscript.

BMJ Open is an open access journal and the full, final, typeset and author-corrected version of record of the manuscript is available on our site with no access controls, subscription charges or pay-per-view fees (http://bmjopen.bmj.com).

If you have any questions on BMJ Open's open peer review process please email editorial.bmjopen@bmj.com

BMJ Open

Evaluating NHS stop smoking service engagement in community pharmacies using simulated smokers: fidelity assessment of a theory-based intervention

Journal:	*BMJ Open*
Manuscript ID	bmjopen-2018-026841
Article Type:	Research
Date Submitted by the Author:	24-Sep-2018
Complete List of Authors:	Jumbe, Sandra; Barts and The London School of Medicine and Dentistry, Centre for Primary Care and Public Health; Queen Mary University of London<br>James, Wai Yee; Barts and The London School of Medicine and Dentistry, Centre for Primary Care and Public Health<br>Madurasinghe, Vichithranie; Barts and The London School of Medicine and Dentistry,<br>Steed, Liz; Barts and The London School of Medicine and Dentistry, Centre for Primary Care and Public Health<br>Sohanpal, Ratna; Queen Mary University of London, Centre for Primary Care and Public Health<br>Yau, Tammy; California Northstate University, Medicine<br>Taylor, Stephanie; Queen Mary University of London, Centre for Primary Care and Public Health<br>Eldridge, Sandra; Queen Mary University of London, Centre for Primary Care and Public Health<br>Griffiths, Chris; Barts and The London School of Medicine and Dentistry,<br>Walton, Robert; Barts and The London School of Medicine and Dentistry, Centre for Health Sciences
Keywords:	EDUCATION & TRAINING (see Medical Education & Training), PRIMARY CARE, PUBLIC HEALTH

SCHOLARONE™
Manuscripts

Article Title: Evaluating NHS stop smoking service engagement in community pharmacies using simulated smokers: fidelity assessment of a theory-based intervention

Sandra Jumbe[1]

Wai Yee James[1]

Vichithranie Madurasinghe[1]

Elizabeth A Steed[1]

Ratna Sohanpal[1]

Tammy Yau[2]

Stephanie JC Taylor[1]

Sandra Eldridge[1]

Chris Griffiths[1]

Robert Walton[1]

[1] Barts and The London School of Medicine and Dentistry, Centre for Primary Care and Public Health, Queen Mary University of London, 58 Turner Street, London E1 2AB. United Kingdom
[2] California Northstate University, 9700 West Taron Drive, Elk Grove, CA 95757 USA

Corresponding author:
Dr Sandra Jumbe
Centre for Primary Care and Public Health
Queen Mary University of London
58 Turner Street
London
E1 2AB
Phone: +442078823448
Fax: +442078822552
Email: s.jumbe@qmul.ac.uk

Keywords: smoking cessation, pharmacies, simulated smokers, fidelity assessment

Word count: 3302

For peer review only

ABSTRACT

Objectives: Smokers are more likely to quit if they use the NHS Stop Smoking Service (SSS). However, community pharmacies experience low service uptake. The Smoking Treatment Optimisation in Pharmacies (STOP) programme aims to address this problem by enhancing staff training using a theory-based intervention. In this repilot study we evaluated intervention fidelity using simulated smokers (actors) to assess smoker engagement and enactment of key intervention components by STOP trained staff.

Design: Observational pilot study.

Settings: Five community pharmacies in North East London with an NHS SSS

Methods: Six actors representative of the east London population, were recruited and trained to complete intervention fidelity assessments. Consenting pharmacy staff from five participating pharmacies received STOP Intervention training. Four weeks after the staff training, the actors visited the participating pharmacies posing as smokers eligible for smoking cessation support. Engagement behaviour by pharmacy staff and enactment of intervention components was assessed using a scoring tool derived from the STOP logic model (scoring range of 0-36), and contemporaneous field notes taken by actors.

Results: 18 of 30 completed assessments were with STOP trained staff (10/18 were counter assistants). Trained staff scored higher on smoker engagement (mean 24.4, SD 9.0) than non-trained staff (16.9, SD 7.8). NHS SSS leaflets (27/30) were the most common smoking cessation materials seen on pharmacy visits. Very few pharmacy staff were seen wearing STOP promotional badges (4/30). Most trained counter staff engaged with smokers using leaflets and a few proactively offered appointments with their cessation advisors. Appropriate use of body language was reported on 26/30 occasions alongside use of key phrases from the STOP training session (n=8).

Conclusions: STOP training may change client engagement behaviour in pharmacy staff and could improve uptake of the NHS SSS. A cluster randomised controlled trial is currently in progress to evaluate effectiveness and cost effectiveness.

Article Summary

Strengths and limitations of the study

- We used simulated clients to test a naturalistic fidelity assessment measuring enactment of a complex intervention to promote use of smoking cessation services in community pharmacies
- The method enables quantitative and qualitative evaluation of pharmacy staff behaviour regarding client engagement by helping determine whether key intervention materials are made available to service users, and rating what staff say and do.
- We found that this method worked well and gave an indication that important elements of the intervention were being enacted in the pharmacies, however lack of comparison data means we cannot necessarily attribute these findings to the STOP intervention training.

For peer review only

- This repilot study shows the mystery shopper method is feasible, and we will be using this in main trial to compare intervention and control pharmacies as part of the process evaluation.

BACKGROUND

Behavioural support for smoking cessation is highly cost-effective in reducing tobacco related morbidity and mortality [1-3]. Behavioural interventions include advice, discussion, and targeted activities aiming to: minimise motivation to smoke; increase resolve not to smoke; facilitate strategies to reduce exposure to smoking cues; improve management of smoking urges; promote smoking cessation medication [4-6]. Rising prominence of evidence-based practice has resulted in increased implementation of behavioural support interventions as part of routine healthcare [7]. One example of this is the NHS Stop Smoking Service (SSS) in the United Kingdom (UK), which offers nicotine replacement therapy (NRT) to smokers trying to quit, alongside weekly consultations [8]. Results indicate that smokers engaged with this service are four times more likely to quit than those using NRT alone [9].

Pharmacies with at least one staff member who has completed National Centre for Smoking Cessation and Training (NCSCT) training are able to deliver the NHS SSS [10]. There is strong evidence for the success of the pharmacy-led SSS in cost-effectiveness and good abstinence rates, endorsing behaviour change training of community pharmacy staff as an effective way of helping people to stop smoking [11]. However, service uptake in pharmacy settings is low [12]. Whilst the recent decrease in smoking prevalence in the UK may be a factor, low uptake may also arise from lack of awareness of pharmacies' public health role [13]. Studies also suggest low pharmacy staff confidence in their ability to deliver such services linked to expectation of negative reactions from customers [13-16].

Existing studies looking at the impact of smoking cessation training for pharmacists suggest a range of benefits including increased levels of counselling [17, 18], improved pharmacist consulting behaviour [19-21], and higher quit rates [19, 22]. Whilst previous interventions have shown benefits, their focus was primarily on the smoking cessation consultation itself rather than initial smoker engagement. The Smoking Treatment Optimisation in Pharmacies (STOP) programme was established to enhance delivery of the NHS SSS [23] by targeting self-efficacy, motivation of pharmacy workers and skills to increase smoker engagement [24]. The STOP intervention does not replace NCSCT training for stop smoking advisors but builds upon and reinforces many of the behaviour change techniques learnt [25]. Importantly STOP also takes an organisational approach by training all pharmacy staff, regardless of stop smoking advisor status to target uptake.

Fidelity of intervention delivery involves assessing the extent to which core, prescribed intervention components are delivered as intended and are received by participants [26]. These two elements are critical for successful translation of evidence-based interventions into practice [26, 27]. Results from fidelity assessments may highlight how the intervention is working and aspects that could be improved. However, fidelity assessment methods are not frequently reported [26-28]. To address receipt of the STOP intervention, this study used simulated smoker-clients to

For peer review only - http://bmjopen.bmj.com/site/about/guidelines.xhtml

assess pharmacy staff performance of intervention skills [28-30]. Specifically, we report on the potential of this method to measure enactment of key elements of the intervention and evaluate impact of STOP training on client engagement into the SSS by pharmacy staff.

METHODS

Study Procedure

Ethical approval for this repilot study was obtained prior to recruitment from the Queen Mary Research Ethics Committee [Reference: QMREC1830a]. Six pharmacies were recruited to this repilot study. Twenty staff members from these participating pharmacies individually gave their consent to attend the STOP Training Intervention (Table 2).

Description of the STOP Training Intervention for Pharmacy staff

The STOP Training Intervention content is summarised in Table 1 which outlines the theories and behaviour change techniques upon which the intervention is based. Training methods have also been published previously in the first pilot study [24]. However, after reviewing reseach findings and participants feedback from the first pilot study, several changes were made in this repilot. Firstly, pilot study participants reported difficulty with training location (central London site) and timing (19:00 to 21:30) [24]. To address these logistical bariers to training attendance in this repilot, training was delivered as a half-day session on a Sunday morning. Ten staff from one pharmacy who were unable to attend the Sunday session were given onsite training instead on a weekday convenient to the pharmacy owner. Secondly, both NCSCT trained and untrained pharmacy staff attended the same session to facilitate shared responsibility and focus on initial client engagement when providing the NHS SSS. This links with the intervention's theoretical assumptions and the behaviour change techniques 'organisation structure' and 'social support (practical)' (see Table 1 and Figure 1). Staff were financially reimbursed for travel expenses with amounts based on participants' usual day rates as recommended in the pilot study. Finally, a WhatsApp group was set up for participating staff as a communication tool and information-sharing platform.

Description of Fidelity assessment Training for Actors

Soon after the STOP intervention training was delivered to pharmacies, the STOP repilot team identified and recruited six actors to assess pharmacy staff performance when engaging with simulated smoker-clients and to record display of smoking cessation materials. The actors were purposefully sampled to represent diverse backgrounds reflecting the east London population [31] thus minimising risk of detection by pharmacy staff (Table 3).

These actors attended a 1.5 hour training session led by the STOP repilot team. During the training, each actor was assigned one of six smoking-related scenarios (Figure 2). Each actor practised their scenario during the training session and received group feedback. The session also involved demonstrating use of the fidelity assessment questionnaire, developed by the STOP repilot team to assess pharmacy staff smoker engagement behaviour (Figure 3). The fidelity assessment involves noting presence of stop smoking related material in the pharmacy environment, then rating the extent to which pharmacy staff build general rapport and conversation related to the NHS SSS with an actor, using a Likert scale (Figure 3). The

For peer review only - http://bmjopen.bmj.com/site/about/guidelines.xhtml

questionnaire has a scoring range of 0-36, where higher ratings indicate better client engagement. Items on the questionnaire were chosen to evaluate outputs of the activities represented in the STOP logic model (Figure 1). Actors could also provide written feedback on the questionnaire; referred to as field notes, to detail poignant aspects of their interaction where necessary. The questionnaire was put onto Qualtrics, an online survey software [32] enabling actors to complete their fidelity assessment electronically after each visit and aiding immediate receipt of source data for the study team.

The actors practised completing the fidelity assessment questionnaire through Qualtrics by observing the trainers role-playing interactions between smokers and pharmacy staff and rating each interaction. In addition, actors were asked to note the name of each pharmacy worker they interacted with by asking directly or looking at their name badge. If this was not possible, they provided a detailed description of the pharmacy worker instead. This enabled the study repilot team to identify simulated interactions that were with pharmacy staff who had attended the STOP intervention training as not all staff in participating pharmacies attended the training.

Fidelity Assessment Pharmacy Visits

Between four and six weeks after the STOP intervention training was delivered to participating pharmacies, each actor visited every pharmacy once using their simulated smoker-client scenario, resulting in 30 completed fidelity assessments (6 actors x 5 pharmacies). Actors were given two weeks to complete their visits although to facilitate a naturalistic approach no fixed schedule was imposed. All participating pharmacies provided written consent for the simulated smoker visits. However, staff were blind to visit timelines and frequency to minimize risk of detection and any associated behaviour change.

Data analysis

Data collected on Qualtrics were analysed using SPSS version 24. Descriptive statistics were used to analyse pharmacy staff recruitment and fidelity assessment outcomes. The qualitative actors' field notes were examined using thematic analysis.

Patient and Public Involvement

Prior to starting this repilot study, a focus group with 4 pharmacy based stop smoking advisors and 2 counter staff was conducted by the study team to further inform barriers and facilitators to pharmacy staff attending the STOP Intervention training.

For peer review only

RESULTS

Smoker engagement ratings

All actors completed their allocated five visits. In total 18 assessments were with pharmacy staff (8 pharmacists or stop smoking advisors, and 10 counter assistants) who had attended the stop training and nine assessments were with pharmacy staff who hand not attended STOP training. On three occasions the training status of the assese was unclear.

Table 4 shows total smoker client engagement scores allocated to each pharmacy interaction. Colours are used to differentiate between interactions with pharmacy staff who attended the STOP intervention training and those who did not. Actors encountered more STOP trained staff in pharmacies with a high staff STOP training attendance. Simulated smoker-clients rated interactions with STOP trained staff higher than interactions with staff who did not attend STOP training (Table 5).

Display of Stop Smoking Materials

Table 6 shows the number of actors (n=6) who reported seeing specific smoking cessation materials during their pharmacy visits. The materials were signposted in the STOP training as useful resources for engaging potential smoker-clients to NHS SSS. At least four or more actors reported seeing NHS SSS leaflets during their pharmacy visit. The majority of actors (n=5) did not see any staff members wearing a STOP badge.

Qualitative analysis of field notes taken by actors

Thematic analysis of actors' field notes provided an indication of the extent of enactment of the STOP Intervention. NHS SSS leaflets were a popular tool for disseminating smoking cessation information. All actors reported being given a leaflet from at least one pharmacy. Some pharmacies provided customised leaflets indicating efforts to deliver a more tailored/personalised service.

> *'I was surprised to be given a leaflet with his name printed on it and the times the pharmacy is open and the leaflet did have smoking cessation clinic on it' (actor 6*)
>
> *'Gave out loads of standard (NHS) leaflets plus one he made up himself' (actor 1)*

Aside from leaflets, actors reported being given key details of the NHS SSS proactively in interactions with STOP trained staff. This was at times despite there being other customers in the pharmacy waiting to be served.

> *'Chemist (said) we can help you if you decide to go on the twelve week course with emotional help and medication' (actor 2)*
>
> *'Stressed that I can come anytime for a consultation with himself, it is free, you can just walk in, takes about 15-20 mins gave me leaflet and took the time to go through the 12 week smoking cessation programme despite the pharmacy being busy' (actor 5)*

There was also evidence of clients being offered a smoking cessation appointment by staff at the counter. Actors found most pharmacy staff were proactive in giving the option to book an appointment to see the smoking cessation specialist. A few actors

For peer review only - http://bmjopen.bmj.com/site/about/guidelines.xhtml

were directed to a private room where they spoke with an advisor about joining the NHS SSS.

'He did mention that it was a 12 week programme and offered to sign me up a few times' (actor 1)

'Very helpful. Offered me the program immediately and nicotine replacement therapy' (actor 6)

'Staff at counter were very eager. It was hard to stop them from referring me to their 'trained' person for a private chat.' (actor 4)

Even when actors declined to join the service, pharmacy staff were keen on giving them smoking cessation leaflets to take home with them or telling clients to come back whenever they felt ready.

'Told me about the 12 week program and when I said I had to leave, said I could come back anytime I felt more ready. Got leaflets too' (actor 4)

These two aspects of giving SSS leaflets and the 'keep the door open' approach were focused on during the training sessions, as several attendees raised the issue of missing potential clients due to the busy pharmacy environment. Throughout group discussions, and role-play exercises, the group felt this approach would particularly help counter staff minimise loss of potential clients needing smoking cessation support.

Several actors described evidence of trained pharmacy staff trying to build rapport by using body language and active listening.

'Good eye contact, very pleasant' (actor 2)

'Very attentive and listened to content in my scenario' (actor 4)

On one occasion, one staff member even shared their personal experience of using the service, suggesting a sense of personal commitment to helping others quit.

'Self-disclosed that she is using the service and has found it amazing' (actor 6)

Actors also reported use of key phrases or facts by pharmacy staff that were covered in the STOP training sessions or circulated on the WhatsApp group during their smoking cessation interactions, demonstrating retention of knowledge from the STOP training intervention.

'Tom was eager to tell me that my son would need to want this himself … he said there would be little or no point if my son didn't want to stop himself' (actor 6)

'70% of smokers want to quit but just need help' (actor 5)

For peer review only - http://bmjopen.bmj.com/site/about/guidelines.xhtml

DISCUSSION

This repilot study shows that the methods we designed using simulated clients to assess fidelity of a complex intervention worked in practice and gives preliminary evidence of enactment of key intervention components. Results suggest that client engagement was better in pharmacy staff who attended training, with improved consulting styles and increased use of intervention materials. Qualitative analysis of contemporaneous field notes taken by actors confirmed the availability and use of some smoking cessation materials. The analysis also suggested that pharmacy staff (including those without NCSCT training) were using consultation skills and appropriate words and phrases which were taught in their STOP intervention training. From a social cognitive theoretical perspective, this infers improved knowledge through vicarious learning and increased self-efficacy to provide basic SSS information to smoker-clients.

Strengths

Simulated clients, commonly known as 'mystery shoppers' are widely used in marketing to measure aspects of customer care. As staff are unaware of the simulated client's identity this provides an opportunity for a naturalistic fidelity assessment of how well knowledge and skills from the intervention were received. Similar methods have been found to be rigorous and robust for measuring practice in this setting [29, 33, 34].

The fidelity assessment questionnaire used by the actors allows for both quantitative and qualitative measurement of pharmacy staff behaviour regarding engagement with smoking cessation services in their working environment. We found that qualitative data from actors' field notes tended to confirm the quantitative ratings.

Our previous research showed that information from the patient coupled with visual and linguistic cues affected advisors' perceptions of the chances of quitting and hence the likelihood of recruitment into the service [15, 16]. Previous studies also confirm that patient characteristics such as age, ethnicity and mental status may form barriers to engaging service users into smoking cessation interventions [15, 35]. Thus we used actors from diverse backgrounds presenting a range of different scenarios. Beliefs and attitudes underlying prejudgements of treatment success were addressed in the STOP training [16, 24]. This repilot study suggests that STOP trained pharmacy staff engaged with all the actors regardless of age and ethnicity and the scenario presented.

The results of this repilot study have informed the STOP logic model in readiness for the STOP trial (Figure 1). Specifically, slight changes to the STOP intervention materials have been made where the flipchart and tar jar have been replaced by a desk calendar which contains a condensed version of the intervention slides. So it works as an information resource with the added working function of a calendar which can be placed at the counter and in the consultation room for both cessation advisors and support staff to use. Site initiation visits and training sessions have

For peer review only - http://bmjopen.bmj.com/site/about/guidelines.xhtml

been separated because they have a unique purpose and need to take place at different times and in different settings. The outputs in the logic model have been revised to reflect the elements of the intervention which are focussed on staff behaviour more clearly.

Weaknesses

Staff members who chose to attend training may have had different baseline characteristics when compared to those that did not attend. We did not assess engagement skills before the intervention to gauge base level of engagement with clients before STOP Training. Nevertheless, our findings are consistent with the suggestion that STOP training may improve pharmacy staff members' ability to engage clients into the NHS SSS.

In the main trial we plan to use the same method of fidelity assessment outlined here. However we will include control pharmacies where staff have not been offered STOP training which will allow us to compare the performance of pharmacy staff who received STOP training and who did not, to quantify the benefit arising from the intervention.

There was inconsistent reporting of SSS materials seen by actors, particularly in rates of display for posters and the STOP study badge. There were instances were in the same pharmacy some actors reported seeing a poster or leaflets whilst others did not. This may reflect human error, in that there could have been posters up that some actors simply did not see because they were distracted by the busy pharmacy environment. Another explanation could be that since the actors visited at different times, the materials were displayed on some days but not on others.

Strengths and weaknesses in the context of other studies

Previous studies using simulated patients to assess community pharmacy staff performance have focused on provision of over the counter medication [29, 34; 36] whereas our research examines the expanded role of pharmacy staff as agents for health behaviour change. However, we used similar methods: covert visits, blinding to time and number of visits to minimise detection [29, 34]. A key strength of our approach was the use of actors with lived experience of smoking or smoking related health conditions such as asthma. Moreover, feedback from staff in our study indicated no detection of our simulated visits whilst detections were reported in other studies [29, 36].

In this study we assessed the initial interaction between pharmacy worker and smoker which usually take place over the counter in community pharmacies. However, other researchers have audio-recorded consultations with stop smoking advisors allowing detailed examination of their interactions with clients which take place in a dedicated /private consulting room [8]. Due to ethical considerations related to obtaining patient consent, it was difficult to audio-record naturalistic interactions with pharmacy users within the time limited setting of this repilot study. In the main trial, we aim to supplement data from simulated smoker-client visits with audiotaped follow-up consultations between stop smoking advisors and actual service users.

For peer review only - http://bmjopen.bmj.com/site/about/guidelines.xhtml

One study [30] with a similar aim and methodological approach to ours randomly assigned pharmacies to two different scenarios [37]. With our method, all pharmacies were assessed against all six scenarios thus examining a broader range of skills and allowing a more thorough evaluation of potential gaps in service delivery. In the main trial we will use a balanced design where each pharmacy is exposed to each scenario which will allow more rigorous comparison of the degree to which the intervention is implemented between different pharmacies.

Implications for clinical practice and policy

The methods that we outline here could potentially be adapted to evaluate the effects of any training programme intended to modify the clinical practice of pharmacy staff. Given that government policy in the UK is to expand the range of clinical services provided in pharmacies [38, 39] methods to evaluate the effects of training may be useful in refining interventions and developing new training programmes.

Unanswered questions and future research

The actors noted adequate display of intervention materials in the pharmacies. However, certain materials were displayed more prominently than others. Therefore in the main trial, there is a need to evaluate carefully the use of intervention materials and to understand reasons why certain materials are given more prominence and used more often than others. A better understanding of these factors may lead to development of more effective intervention materials which are more likely to be available to potential users of the intervention.

Author Contributions

SJ led development of the final version of the intervention and designed the fidelity assessment. WYJ and SJ recruited sites, delivered the training, and collected qualitative and quantitative data for the repilot study. SJ, VM and TY analysed the data. ES led development of the initial intervention for the 1st pilot study, and gave critical comments on the manuscript alongside WYJ, VM, TY, ST and SE. SJ and RW drafted the paper with input from all other authors. All authors have seen and approved the final manuscript.

Acknowledgments

Mr Darush Attar who is a pharmacist, behaviour change specialist and national public health trainer specialised in smoking cessation, co-facilitated the STOP Intervention training with SJ and WYJ. This work has been previously presented as a thematic poster at the European Respiratory Society Milan 2017 International Congress.

Funding

This is a repilot study of the NIHR-funded STOP programme on which RW is chief investigator and CG, SE, ST and TG are co-investigators. NIHR Programme grant RP-PG-0609-10181.

Competing interests None declared.

For peer review only

Ethics approval Ethical approval for the study was obtained from the Queen Mary Research Ethics Committee [Reference: QMREC1830a]

Provenance and peer review Not commissioned; externally peer reviewed.

Data sharing statement Study data (with identifying information removed) will be available, following completion of the project, on request from the study guarantor, Robert Walton, r.walton@qmul.ac.uk.

REFERENCES

1. Lancaster T, Stead LF: Individual behavioural counselling for smoking cessation. *Cochrane Database Syst Rev* 2005:CD001292.

2. Stead LF, Lancaster T: Group behaviour therapy programmes for smoking cessation. *Cochrane Database Syst Rev* 2005:CD001007.

3. Stead L, Perera R, Lancaster T: Telephone counseling for smoking cessation. *Cochrane Database Syst Rev* 2006, 2005, CD002850.

4. Michie S, Churchill S, West R: Identifying evidence-based competences required to deliver behavioural support for smoking cessation. *Ann Behav Med* 2011, 41(1):59–70.

5. West R, Stapleton J: Clinical and public health significance of treatments to aid smoking cessation. *Eur Respir Rev* 2008, 17(110):199–204.

6. Lorencatto F, West R, Michie S: Identifying evidence-based behaviour change techniques for smoking cessation in pregnancy. *Nicotine & Tobacco Research* 2012, 14(9):1019–1026. doi:10.1093/ntr/ntr324.

7. Raw M, Regan S, Rigotti NA, McNeill A: A survey of tobacco dependence treatment services in 36 countries. *Addiction* 2009, 104(2):279–87.

8. Lorencatto F, West R, Christopherson C, Michie S. Assessing fidelity of delivery of smoking cessation behavioural support in practice. *Implementation Science*. 2013; 8(1):40.

9. Bauld L, Bell K, McCullough L, Richardson L, Greaves L. The effectiveness of NHS smoking cessation services: a systematic review. Journal of Public Health. 2010 Mar 1;32(1):71-82.

10. Brose LS, West R, Michie S, Kenyon JA, McEwen A. Effectiveness of an online knowledge training and assessment program for stop smoking practitioners. Nicotine & Tobacco Research. 2012 Jan 16;14(7):794-800

11. Anderson C, Blenkinsopp A & Armstrong M, 2009. The contribution of community pharmacy to improving the public's health: summary report of the literature review 1990–2007. Five earlier reports at PharmacyHealthLink.

For peer review only

12. Latif A. & Boardman H. Community pharmacists' attitudes towards medicines use reviews and factors affecting the numbers performed. *Pharm World Sci*, 2008; 30:536–543

13. Saramunee K, Krska J, Mackridge A, Richards J, Suttajit S, Phillips-Howard P. How to enhance public health service utilization in community pharmacy?: General public and health providers' perspectives. Research in Social and Administrative Pharmacy. 2014;10(2):272-84.

14. Eades CE, Ferguson JS, O'Carroll RE. Public health in community pharmacy: a systematic review of pharmacist and consumer views. BMC public health. 2011;11(1):582.

15. Hudmon KS, Prokhorov AV, Corelli RL. Tobacco cessation counseling: pharmacists' opinions and practices. Patient education and counseling. 2006 Apr 30;61(1):152-60.

16. Sohanpal R, Rivas C, Steed L, MacNeill V, Kuan V, Edwards E, Griffiths C, Eldridge S, Taylor S, Walton R. Understanding recruitment and retention in the NHS community pharmacy stop smoking service: perceptions of smoking cessation advisers. BMJ open. 2016 Jul 1;6(7):e010921.

17. Sinclair H, Bond C, Lennox, AS, et al. An evaluation of a training workshop for pharmacists based on the Stages of Change model of smoking cessation. *Health Education Journal* 1997;56:296-312.

18. Sinclair HK, Bond CM, Lennox AS, et al. Training pharmacists and pharmacy assistants in the stage-of-change model of smoking cessation: a randomised controlled trial in Scotland. *Tob Control* 1998;7(3):253-61.

19. Maguire TA, McElnay JC, Drummond A. A randomized controlled trial of a smoking cessation intervention based in community pharmacies. *Addiction* 2001;96(2):325-31. doi: 10.1080/09652140020021062

20. Greenhalgh T, Macfarlane F, Steed L, Walton R. What works for whom in pharmacist led smoking cessation support: realist review. BMC Medicine 2016 14:209. DOI: 10.1186/s12916-016-0749-5

21. Rivas C, Sohanpal R, McNeill V,Steed L, Edwards E, Griffiths C, Eldridge S, Taylor SJC, Walton R. Defining effective communication strategies in the NHS community pharmacy stop smoking programme: a naturalistic, matched-pairs analysis of smoker consultations. BMJ Open 2017;7:e015664. doi: 10.1136/bmjopen-2016-015664

22. Bock BC, Hudmon KS, Christian J, Graham AL, Bock FR. A tailored intervention to support pharmacy-based counseling for smoking cessation. Nicotine & tobacco research. 2010 Jan 25;12(3):217-25.

For peer review only - http://bmjopen.bmj.com/site/about/guidelines.xhtml

23. Madurasinghe VW, Sohanpal R, James W-Y, Steed L, Eldridge SE, Taylor SJC, Griffiths CJ, Walton R. Smoking treatment optimisation in pharmacies (STOP): a cluster randomised pilot trial of a training intervention. Pilot and Feasibility Studies 2017:3:1 DOI: 10.1186/s40814-016-0120-9

24. Steed L, Sohanpal R, James WY, Rivas C, Jumbe S, Chater A, Todd A, Edwards E, MacNeill V, Macfarlane F, Greenhalgh T. Equipping community pharmacy workers as agents for health behaviour change: developing and testing a theory-based intervention. BMJ Open. 2017 Mar 22.

25. West R, Walia A, Hyder N, et al. Behavior change techniques used by the English Stop Smoking Services and their associations with short-term quit outcomes. *Nicotine Tob Res*
2010;12(7):742-7. doi: 10.1093/ntr/ntq074

26. Breitenstein SM, Gross D, Garvey CA, Hill C, Fogg L, Resnick B. Implementation fidelity in community-based interventions. Research in nursing & health. 2010 Apr 1;33(2):164-73.

27. Boutron I, Moher D, Altman D, Scultz K, Ravaud P: Extending the CONSORT statement to randomized trials of Non-pharmacologic treatment: explanation and elaboration. Annals of Internal Medicine 2008, 148:295–309

28. Rixon L, Baron J, McGale N, Lorencatto F, Francis J, Davies A. Methods used to address fidelity of receipt in health intervention research: a citation analysis and systematic review. BMC health services research. 2016 Dec;16(1):663.

29. Watson MC, Skelton JR, Bond CM, Croft P, Wiskin CM, Grimshaw JM, Mollison J. Simulated patients in the community pharmacy setting–Using simulated patients to measure practice in the community pharmacy setting. Pharmacy World and Science. 2004 Feb 1;26(1):32-7.

30. Saba M, Diep J, Bittoun R, Saini B. Provision of smoking cessation services in Australian community pharmacies: a simulated patient study. International journal of clinical pharmacy. 2014 Jun 1;36(3):604-14.

31. Robson J, Dostal I, Madurasinghe V, Sheikh A, Hull S, Boomla K, Page H, Griffiths C, Eldridge S. The NHS Health Check programme: implementation in east London 2009–2011. BMJ open. 2015 Apr 1;5(4):e007578.

32. Snow J, Mann M. Qualtrics Survey Software: Handbook for Research Professional. Provo, UT: Qualtrics Labs.

33. Watson MC, Norris P, Granas AG. A systematic review of the use of simulated patients and pharmacy practice research. International Journal of Pharmacy Practice. 2006 Jun 1;14(2):83-93.

34. Watson MC, Cleland JA, Bond CM. Simulated patient visits with immediate feedback to improve the supply of over-the-counter medicines: a feasibility study. Family practice. 2009 Oct 14;26(6):532-42.

For peer review only

35. Williams DM, Newsom JF, Brock TP. An evaluation of smoking cessation-related activities by pharmacists. Journal of the American Pharmaceutical Association (1996). 2000 Mar 1;40(3):366-70.

36. Weiss MC, Booth A, Jones B, Ramjeet S, Wong E. Use of simulated patients to assess the clinical and communication skills of community pharmacists. Pharmacy world & science. 2010 Jun 1;32(3):353-61.

37. Brose LS, West R, Michie S, et al. Evaluation of Face-to-Face Courses in Behavioural Support for Stop Smoking Practitioners. Journal of Smoking Cessation 2012;7:25-30. doi:doi:10.1017/jsc.2012.6

38. Donovan GR, Paudyal V. England's Healthy Living Pharmacy (HLP) initiative: Facilitating the engagement of pharmacy support staff in public health. Research in social and administrative pharmacy. 2016 Mar 1;12(2):281-92.

39. Twigg MJ, Wright D, Kirkdale CL, Desborough JA, Thornley T. The UK Pharmacy Care Plan service: Description, recruitment and initial views on a new community pharmacy intervention. PloS one. 2017 Apr 3;12(4):e0174500.

For peer review only

Tables

Table 1: Detailed description of the STOP Intervention

For peer review only

PHARMACY SITE INITIATION VISIT	Content	Theoretical Basis	Behaviour change techniques
	Explain the study to pharmacist in charge or manager.<br>Mention potential revenue stream from smoking cessation.<br><br>Emphasise to staff how this fits well with their wider role in health promotion.<br><br>Raise awareness in all staff in preparation for invitation to training.<br><br>Communicate the advantages of the STOP intervention over usual practice, i.e. it is brief and show how it fits with overall 'pharmacy' identity.<br><br>Address pre implementation concerns.<br><br>Provide financial incentive for attending training (only received on completion of training)<br><br>Emphasise backing from local and national opinion leaders and organisations (e.g. Local Pharmaceutical Committee, Royal Pharmaceutical Society, local CCGs and public health commissioners	Adoption by individuals: concerns in preadoption stage (DIT)<br><br>The innovation: compatibility;<br>relative advantage; low complexity (DIT)<br><br>Outer context: incentives (DIT)<br><br>Diffusion and dissemination: opinion leaders (DIT)	10.2 Material reward (behaviour)<br><br>9.1 Credible source<br><br>1.2 Problem Solving<br><br>6.3 Information about others' approval
**TRAINING SESSION**	**Content**	**Theoretical Basis**	**Behaviour change techniques**
**Introduction**	General orientation to STOP programme and aims of training.<br><br>Emphasise backing from local and national opinion leaders and organisations (e.g. Local Pharmaceutical Committee, Royal Pharmaceutical Society, local CCGs and public health commissioners<br><br>Discussion of impact of advisor behaviour on client stop smoking outcomes so far and health benefits to patients from stopping smoking.<br><br>Delivered in mixed groups of pharmacists and other pharmacy workers to promote cohesive working practices within the individual pharmacies.	Outcome Expectancies (SCT)<br><br>Diffusion and dissemination: opinion leaders (DIT)<br><br>Implementation and routinisation: organizational structure (DIT)	5.1 Information on health consequences of behaviour<br><br>9.1 Credible source<br><br>10.6 Nonspecific incentive<br><br>15.1 Verbal persuasion about capability

For peer review only - http://bmjopen.bmj.com/site/about/guidelines.xhtml

**Why are we here?**	Smoking facts and exploration of motivation for helping smokers to quit with feedback.<br><br>Discuss focus on pharmacy setting, emphasising the non-medication related, professional and public health aspects of the pharmacy role.<br><br>Does engaging and supporting smokers' quit fit with role identity, any barriers? Encourage self-perception as supporters and providers of health, how one will feel if help smokers quit.<br><br>Emphasise the non-medication related, professional and public health aspects of the pharmacy role, promote a person-centred rather than product-centred ethos and foster a strong sense of professionalism	Intrinsic and extrinsic motivators (SDT)<br><br>The innovation: compatibility (DIT)	5.6 Information about emotional consequences<br><br>9.2 Pros and cons<br><br>6.3 Information about others approval<br><br>13.1 Identification of self as a role model<br><br>15.3 Focus on past success
**Engaging Clients**	Celebrate successful cases.<br><br>Group exercise and discussion on difficult and easy clients to engage – potential problems and solutions.<br><br>Addressing pharmacy workers beliefs and attitudes e.g. prejudgement of success or failure.	Self-efficacy (SCT)<br>Modelling (SCT)<br>Vicarious learning (SCT)	1.2 Problem solving,<br>8.1 Behavioural practice and rehearsal<br>9.2 Pros and cons<br>13.3 Incompatible beliefs<br>15.3 Focus on past success
**Patient-centred approach: Building rapport & shift of focus**	Introduction of patient centred approach using group exercise. Group identification and discussion of the importance of utilising basic communication skills (rapport, active listening, questioning).<br><br>Review how patient-centred approach can be incorporated into smoking cessation interactions for better patient outcomes<br><br>Role-play demonstration with senior pharmacist, participant practice. How to maximise opportunity with environmental resources e.g. staff wearing STOP badges to prompt client interaction, STOP posters<br><br>Emphasise predictable improved results, simplicity of use and benefits over usual practice.	Self-efficacy (SCT)<br>Modelling (SCT)<br>Vicarious learning (SCT)<br>The innovation: relative advantage; compatibility; low complexity (DIT)	4.1 Instruction on performance of behaviour,<br>6.1 Demonstration of behaviour<br>7.1 Prompts and cues<br>8.1 Behavioural practice and rehearsal<br>8.2 Behaviour substitution<br>8.6 Generalisation of target behaviour<br>9.2 Pros and cons<br>9.3 Comparative imagining of future outcomes
**NCSCT Knowledge**	Review group's NCSCT knowledge with a quiz and general	Self-efficacy (SCT)	1.6 Discrepancy between

For peer review only - http://bmjopen.bmj.com/site/about/guidelines.xhtml

**Review**	feedback		current behaviour and goal<br>9.1 Credible source
**Pharmacy role in smoking cessation**	Discuss individual pharmacies' NHS Stop Smoking service structure and purpose of smoking treatment, with experienced advisers sharing current and best practice. Group reflection on challenges	Homophily (DIT)	1.7 Review outcome goal(s)
**Behaviour change as smoking cessation treatment**	Emphasise behaviour change support as part of smoking cessation treatment within NHS SSS.<br><br>Information on how to assess someone's readiness to quit smoking using 1-10 scales.	Self-efficacy (SCT)<br>Self-regulation (SCT)<br>The innovation: compatibility (DIT)	4.1 Instruction how to perform behaviour<br>8.1 Behavioural practice and rehearsal
**Behaviour change using patient centred approach in Smoking Cessation Treatment: Double Whammy**	Brainstorm factors that influence behaviour change – (role of beliefs, capability, opportunity alongside knowledge)<br><br>How to elicit individuals' motivations, barriers and potential strategies to change behaviour versus offering solutions. Using 'What else questions'.<br><br>Understanding the 'non-smoker identity' and how to communicate to client<br><br>Demonstration & Role play<br><br>What makes this client centred approach difficult - advantages, disadvantages, barriers and strategies to aid implementation	Outcome Expectancies (SCT)<br>Modelling (SCT)<br>Self-efficacy (SCT)<br>The innovation: fuzzy boundaries (DIT)	1.2 Problem Solving<br>4.1 Instruction on how to perform behaviour<br>6.1 Demonstration of behaviour<br>8.1 Behavioural practice and rehearsal<br>9.2 Pros and Cons
**Client engagement in pharmacy settings: Planning a Quit & Dealing with Lapses**	Discuss planning a quit and how to help people make a specific plan using a SMART approach. Go over ways to discuss with lapses and provide supportive praise.<br><br>Discussion of how to talk about willpower and the role of the open door.<br><br>Watch and reflect on video of strong and weak consultations of quit planning.<br><br>Demonstration & Role play	Modelling (SCT)<br><br>Self-efficacy (SCT)	1.1 Goal setting (behaviour)<br>1.2 Problem solving<br>3.1 Social support (unspecified)<br>4.1 Instruction on how to perform behaviour<br>6.1 Demonstration of behaviour<br>8.1 Behavioural practice and rehearsal

For peer review only - http://bmjopen.bmj.com/site/about/guidelines.xhtml

**Client engagement in pharmacy settings: Goal Setting & Making a Commitment**	Facilitate goal setting and elicit verbal commitment from participants.<br><br>Demonstration (via video)<br><br>Practice cohesive working amongst trainees through role play using multiple scenarios and observer feedback	Modelling (SCT)	1.1 Goal Setting (behaviour)<br>1.9 Commitment<br>3.1 Social support (unspecified)<br>8.1 Behavioural practice and rehearsal<br>15.1 Verbal persuasion about capability
**Implementing STOP**	Review how to implement STOP in practice (i.e. prompts and WhatsApp support) by facilitating discussion of implementation plans alongside facilitators or barriers with pharmacy team<br><br>Highlight use of local champions and prompts/cues including the Double Whammy (a desk calendar with visual cues and example questions to ask) to prompt client interaction.<br><br>Highlight ongoing social support via WhatsApp<br>Promote adaptation of non-core elements of the intervention through a prompted pharmacy team meeting to discuss implementation of the intervention according to the needs of each individual pharmacy e.g. appointment of individual champions, monthly 'STOP' smoking days	Self-regulation (SCT)<br>Intrinsic/Extrinsic Motivators (SDT)<br>The innovation: augmentation/support (DIT)<br>The innovation: trialability; reinvention; fuzzy boundaries; champions (DIT)<br><br>Implementation & routinisation: organisational structure (DIT)	4.1 Instruction on how to perform behaviour<br>1.2 Problem Solving<br>7.1 Prompts and Cues<br>3.2 Social support (practical)<br>1.4 Action planning<br><br>10.1 Material incentive (behaviour)
**End of Session**	Participants provided with a certificate for attending the training linked to CPD (endorsed by RPS)<br><br>Provide financial reward for those who have completed intervention training	Outer context: incentives (DIT)	10.2 Material reward
**6 WEEK BOOSTER VISIT**	**Content**	**Theoretical Basis**	**Behaviour change techniques**
**Feedback on smoker engagement and update on STOP implementation**	Identification of organizational barriers, facilitators to implementing STOP in individual pharmacies. Facilitating action plans to implement STOP in their pharmacy.<br><br>Any further thoughts on how the intervention can be adapted to local circumstances?<br><br>Review pharmacy staffs' self-efficacy of skills<br><br>Troubleshoot based on performance feedback, assessment by mystery shoppers* and staff's self-reported self-efficacy	The innovation: fuzzy boundaries (DIT)<br>Adoption by individuals: concerns in preadoption stage (DIT)<br><br>The innovation: reinvention<br><br>The innovation: augmentation/support (DIT)	1.1 Goal Setting<br>1.2 Problem solving<br>1.4 Action Planning<br>1.5 Review behaviour goal(s)<br>1.6 Discrepancy between current behaviour and goal<br>1.7 Review outcome goal<br>1.9 Commitment<br><br>2.2 Feedback on behaviour<br>2.7 Feedback on outcome of

For peer review only - http://bmjopen.bmj.com/site/about/guidelines.xhtml

	*Mystery shoppers are trained actors who approach staff in intervention pharmacies using smoking-related scenarios to assess client engagement and evaluate presence of STOP smoking environmental cues (posters, badges)	Self-efficacy (SCT)<br><br>The innovation: trialability (DIT)	behaviour<br><br>7.1 Prompts and cues<br><br>8.3 Habit formation

Table 2: Pharmacy Staff Demographics

CHARACTERISTICS		SUPPORT STAFF (n=16)	STOP SMOKING ADVISORS (n=4)	Total
Mean age in years (Range)		29 (16-49)	37 (30-54)	30 (16-54)
Male (%)		38	100	50
Graduate or higher (%)		25	100	40
Never smoked (%)		75	100	80
Job titles	**Counter Assistant**	10		
	**Dispensing Chemist**	4		
	**Trainee pharmacist**	2		
	**Pharmacist**		2	
	**Pharmacist technician**		1	
	**Business Manager**		1	

Table 3: Actor demographics

Actor ID	Age	Gender	Ethnicity	Education	Smoking Status
01	49	Male	White	Postgraduate	Never

For peer review only - http://bmjopen.bmj.com/site/about/guidelines.xhtml

					smoked
02	56	Female	White British	Other	Ex-smoker
03	54	Male	Mixed	Graduate	Ex-smoker
04	32	Male	Black	Graduate	Never smoked
05	22	Female	Mixed	NVQ L3	Ex-smoker
06	58	Female	White British	Professional (CPCAB)	Ex-smoker

For peer review only

Table 4: Smoker engagement ratings from simulated smokers

		Client Engagement Ratings by Actor					
		**1**	**2**	**3**	**4**	**5**	**6**
**S06**	**100**	36	34	36	34	34	29
**S02**	**60**	29	14	18	29	6	14
**S01**	**57**	18	14	16	25	27	6
**S05**	**40**	10	4	20	15	28	14
**S03**	**33**	14	24	25	27	22	20
PHARMACY SITE	***% of pharmacy staff trained***	**LEGEND**<br>☐ Completed STOP training<br>■ Did not attend training<br>▩ Unable to identify					

For peer review only - http://bmjopen.bmj.com/site/about/guidelines.xhtml

Table 5: Mean Client Engagement Scores for trained *v* untrained staff

	ATTENDED STOP TRAINING	N	Mean	Std. Dev.
OVERALL CLIENT ENGAGEMENT	No	12	16.9	7.7
	Yes	18	24.4	9.0
BUILDING RAPPORT	No	12	6.7	3.7
	Yes	18	9.1	2.3
CONVERSATION	No	12	10.3	5.1
	Yes	18	15.3	7.1

For peer review only

Table 6: Display of smoking cessation materials

Pharmacy site	NHS SSS Poster	NHS SSS Leaflet	STOP study poster	STOP study badge
**S01**	2	4	0	0
**S02**	1	6	3	0
**S03**	3	6	3	3
**S05**	2	5	1	0
**S06**	3	6	3	1

For peer review only - http://bmjopen.bmj.com/site/about/guidelines.xhtml

Figures

Figure 1: STOP Programme Final Logic Model

Figure 2: Brief scenario outlines

Figure 3: Fidelity Assessment Questionnaire

For peer review only

For peer review only - http://bmjopen.bmj.com/site/about/guidelines.xhtml

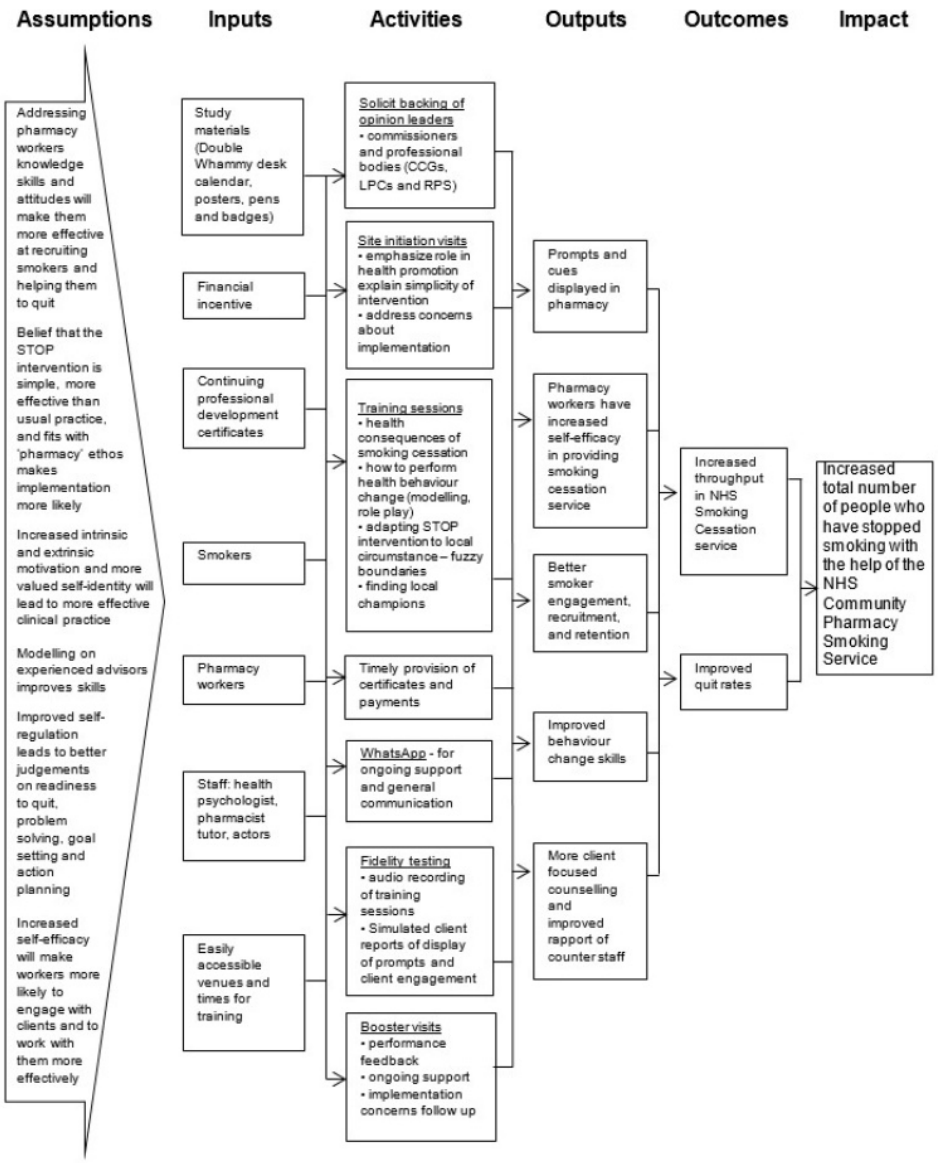

264x336mm (72 x 72 DPI)

Figure 2: Brief scenario outlines

**Scenario 1**<br>Individual picks up SSS visual resource and reads through, attends counter and asks if the pharmacy offers advice on how to stop smoking. Has discovered teenage child smokes and wants them to stop.<br><br>Actor required: middle aged woman, white	**Scenario 2**<br>Individual attends counter coughing, with cigarettes obviously on display e.g. holding in hand and putting on counter. Individual tells pharmacy staff they have asthma and need advice on how to stop smoking.<br><br>Actor required: middle aged male, white	**Scenario 3**<br>Individual attends counter with non-smoking related request e.g. purchase pregnancy vitamins. Has cigarettes visible and obviously on display e.g. holding in hand and putting on counter.<br><br>Actor required: middle aged male, ethnic minority
**Scenario 4**<br>Individual attends counter wheezing, with asthma pump e.g. holding in hand and putting on counter. Individual tells pharmacy staff they have asthma and COPD but still smoke. Asthma is getting worse so they need advice on how to stop smoking.<br><br>Actor required: middle aged female, white	**Scenario 5**<br>Individual attends the pharmacy counter, complaining of a sore throat and need of lozenges. Explains that they went partying last night with friends and was smoking so maybe sore throat is linked to that. Friend and mother nagging that sore throat is due to smoking. Ask pharmacy worker what they think it could be?<br><br>Actor required: young female, ethnic minority	**Scenario 6**<br>Individual attends counter, complaining of stress from girlfriend/mum to stop smoking because he has been getting sore throats a lot lately. Not sure if he wants to quit but thought he should check out what the pharmacy have to offer. Saw a sign that says pharmacy has a Stop Smoking Service.<br><br>Actor required: young male, ethnic minority

405x166mm (72 x 72 DPI)

Date: DD / MM / YEAR	Time: ___:___ hours	Scenario number:	Pharmacy ID

Environment

Were the following visible within the pharmacy:-	***Tick if seen***
NHS Stop Smoking Service poster or audiovisual information	
NHS Stop Smoking Service leaflets	
STOP Study Poster	
Staff wearing STOP Study Badges	

Rapport

To what extent did the pharmacy worker demonstrate:- (circle a number)

a) good body language e.g. eye contact, attentiveness 0 1 2 3
b) good listening skills e.g. focused on conversation 0 1 2 3
c) use of open questions 0 1 2 3
d) picking up on client's verbal or visual cues 0 1 2 3

Conversation

To what extent did the pharmacy worker:- (circle a number)

a) Initiate conversation of smoking in response to a verbal or visual prompt 0 1 2 3
b) Raise smoking directly e.g. do you smoke 0 1 2 3
c) Raise smoking indirectly e.g. by talking about second hand smoke 0 1 2 3
d) Tell you there is a smoking cessation service available in the pharmacy 0 1 2 3
e) Highlight that the service is free aside from NRT if pay for prescriptions 0 1 2 3
f) Highlight that people who use the service are 4 times more likely to quit 0 1 2 3
h) Ask you whether you want a referral to the service 0 1 2 3
i) Close conversation by saying you can come back anytime for help/support 0 1 2 3

Additional comments:	Total score:

263x321mm (72 x 72 DPI)

BMJ Open

Evaluating NHS stop smoking service engagement in community pharmacies using simulated smokers: fidelity assessment of a theory-based intervention

Journal:	*BMJ Open*
Manuscript ID	bmjopen-2018-026841.R1
Article Type:	Research
Date Submitted by the Author:	21-Dec-2018
Complete List of Authors:	Jumbe, Sandra; Barts and The London School of Medicine and Dentistry, Centre for Primary Care and Public Health; Queen Mary University of London<br>James, Wai Yee; Barts and The London School of Medicine and Dentistry, Centre for Primary Care and Public Health<br>Madurasinghe, Vichithranie; Barts and The London School of Medicine and Dentistry,<br>Steed, Liz; Barts and The London School of Medicine and Dentistry, Centre for Primary Care and Public Health<br>Sohanpal, Ratna; Queen Mary University of London, Centre for Primary Care and Public Health<br>Yau, Tammy; California Northstate University, Medicine<br>Taylor, Stephanie; Queen Mary University of London, Centre for Primary Care and Public Health<br>Eldridge, Sandra; Queen Mary University of London, Centre for Primary Care and Public Health<br>Griffiths, Chris; Barts and The London School of Medicine and Dentistry,<br>Walton, Robert; Barts and The London School of Medicine and Dentistry, Centre for Health Sciences
**Primary Subject Heading**:	Smoking and tobacco
Secondary Subject Heading:	Health services research, Public health, Research methods
Keywords:	Smoking cessation, community pharmacies, fidelity assessment, simulated patients

SCHOLARONE™
Manuscripts

Article Title: Evaluating NHS stop smoking service engagement in community pharmacies using simulated smokers: fidelity assessment of a theory-based intervention

Sandra Jumbe[1]

Wai Yee James[1]

Vichithranie Madurasinghe[1]

Elizabeth A Steed[1]

Ratna Sohanpal[1]

Tammy Yau[2]

Stephanie JC Taylor[1]

Sandra Eldridge[1]

Chris Griffiths[1]

Robert Walton[1]

[1] Barts and The London School of Medicine and Dentistry, Centre for Primary Care and Public Health, Queen Mary University of London, 58 Turner Street, London E1 2AB. United Kingdom
[2] California Northstate University, 9700 West Taron Drive, Elk Grove, CA 95757 USA

Corresponding author:
Dr Sandra Jumbe
Centre for Primary Care and Public Health
Queen Mary University of London
58 Turner Street
London
E1 2AB
Phone: +442078823448
Fax: +442078822552
Email: s.jumbe@qmul.ac.uk

Keywords: smoking cessation, pharmacies, simulated smokers, fidelity assessment

Word count: 3890

For peer review only

ABSTRACT

Objectives: Smokers are more likely to quit if they use the NHS Stop Smoking Service (SSS). However, community pharmacies experience low service uptake. The Smoking Treatment Optimisation in Pharmacies (STOP) programme aims to address this problem by enhancing staff training using a theory-based intervention. In this study we evaluated intervention fidelity using simulated smokers (actors) to assess smoker engagement and enactment of key intervention components by STOP trained staff.

Design: Observational pilot study.

Settings: Five community pharmacies in North East London with an NHS SSS

Methods: Six actors, representative of East London's population, were recruited and trained to complete intervention fidelity assessments. Consenting pharmacy staff from five participating pharmacies received STOP Intervention training. Four weeks after the staff training, the actors visited the participating pharmacies posing as smokers eligible for smoking cessation support. Engagement behaviour by pharmacy staff and enactment of intervention components was assessed using a scoring tool derived from the STOP logic model (scoring range of 0-36), and contemporaneous field notes taken by actors.

Results: 18 of 30 completed assessments were with STOP trained staff (10/18 were counter assistants). Mean score for smoker engagement was 24.4, (SD 9.0) points for trained and 16.9 (SD 7.8) for untrained staff, respectively. NHS SSS leaflets (27/30) were the most common smoking cessation materials seen on pharmacy visits. Most trained counter staff engaged with smokers using leaflets and a few proactively offered appointments with their cessation advisors. Appropriate use of body language was reported on 26/30 occasions alongside use of key phrases from the STOP training session (n=8). Very few pharmacy staff wore STOP promotional badges (4/30).

Conclusions: STOP training may change client engagement behaviour in pharmacy staff and could improve uptake of the NHS SSS. A cluster randomised controlled trial is currently in progress to evaluate its effectiveness and cost effectiveness.

Article Summary

Strengths and limitations of the study

- We used simulated clients for a naturalistic fidelity assessment measuring enactment of a complex intervention to promote use of smoking cessation services in community pharmacies
- The method enables quantitative and qualitative evaluation of pharmacy staff behaviour regarding client engagement by assessing whether key intervention materials are made available to service users, and rating what staff say and do.
- We found that this method worked well and gave an indication that important elements of the intervention were being enacted in the pharmacies, however lack of comparison data means we cannot necessarily attribute these findings to the STOP intervention training.
- This study shows the simulated client assessment method is feasible, and we will use this in the main trial to compare intervention and control pharmacies as part of the process evaluation.

For peer review only

BACKGROUND

Behavioural support for smoking cessation is highly cost-effective in reducing tobacco related morbidity and mortality [1-3]. Behavioural interventions include advice, discussion, and targeted activities aiming to: minimise motivation to smoke; increase resolve not to smoke; facilitate strategies to reduce exposure to smoking cues; improve management of smoking urges; promote smoking cessation medication [4-6]. Rising prominence of evidence-based practice has resulted in increased implementation of behavioural support interventions as part of routine healthcare [7]. One example of this is the NHS Stop Smoking Service (SSS) in the United Kingdom (UK), which offers smoking cessation treatment including nicotine replacement therapy (NRT) to smokers trying to quit, alongside weekly consultations [8]. Results indicate that smokers engaged with this service are four times more likely to quit than those using NRT alone [9].

Pharmacies with at least one staff member who has completed National Centre for Smoking Cessation and Training (NCSCT) training, often known as a stop smoking advisor (SSA), are able to deliver the NHS SSS [10]. There is strong evidence for the success of the pharmacy-led SSS in cost-effectiveness and good abstinence rates, endorsing behaviour change training of community pharmacy staff as an effective way of helping people to stop smoking [11]. However, service uptake in pharmacy settings is low [12]. Whilst the recent decrease in smoking prevalence in the UK may be a factor, low uptake may also arise from lack of awareness of pharmacies' public health role [13]. Studies also suggest low pharmacy staff confidence in their ability to deliver such services linked to expectation of negative reactions from customers [13-16].

Previous studies looking at the impact of smoking cessation training for pharmacists suggest a range of benefits including increased levels of counselling [17, 18], improved pharmacist consulting behaviour [19-21], and higher quit rates [19, 22]. Whilst previous interventions have shown benefits, their focus was primarily on the smoking cessation consultation itself rather than initial smoker engagement. The Smoking Treatment Optimisation in Pharmacies (STOP) programme was established to enhance delivery of the NHS SSS [23] by targeting self-efficacy, motivation of pharmacy workers and skills to increase smoker engagement [24].

This theory-based complex intervention was developed and tested for acceptability in twelve community pharmacies from three east London boroughs in an initial pilot study [24]. Specifically, 20 SSAs from these pharmacies attended two skills-based training sessions focused on communication and behaviour change skills. Study results confirmed the acceptability of the STOP intervention in terms of overall structure and face-to-face training content, with some participants reporting use of newly learnt skills in practice. However, organisational barriers such as limited finances to cover pharmacist absence and poor acceptability of training venue and times limited pharmacy staff attendance. Another key finding was that very few staff members working at the pharmacy counter attended STOP training sessions. This limited effective service delivery because they were less able to engage in smoking-related conversation with clients [24].

To address logistical barriers highlighted in the first pilot study for attending training, the study team conducted a focus group with four pharmacy-based SSAs and two

For peer review only

counter staff. Based on feedback from this group several changes were made to the intervention to enhance attendance and intervention uptake. Firstly, training delivery was changed from two sessions to a half-day session on a Sunday morning with an option for onsite training on a weekday if more convenient to the pharmacy owner. Secondly, we took an organisational approach training all pharmacy staff (both NCSCT trained and untrained) within the same session to facilitate shared responsibility and focus on initial client engagement. The refined STOP Training Intervention content is summarised in Table 1 which outlines the theories and behaviour change techniques [24, 25] upon which the intervention is based. The STOP logic model describing the programme theory for the intervention is shown in Figure 1.

To address receipt of the refined STOP intervention, we conducted a second pilot in November 2016. The focus of this second pilot study was to assess the fidelity of the refined STOP intervention, that is, to evaluate pharmacy staff performance of intervention skills [28-30] in practice. Fidelity of intervention delivery involves assessing the extent to which core, prescribed intervention components are delivered as intended and are received by participants [26]. These two elements are critical for successful translation of evidence-based interventions into practice [26, 27]. Results from fidelity assessments may highlight how the intervention is working and aspects that could be improved. However, fidelity assessment methods are not frequently reported [26-28]. In this study we piloted the use of the 'simulated client' method as a way of assessing fidelity of the refined STOP intervention, with a focus on how well pharmacy staff engage with potential SSS clients [29-30]. Specifically, this paper reports on the potential of this method, to measure enactment of key elements of the intervention and evaluate impact of STOP training on client engagement into the SSS by pharmacy staff.

METHODS

Study Design

This was an observational pilot study conducted in North East London Boroughs where simulated clients were used to examine how community pharmacies engage with pharmacy users asking for smoking cessation advice. We trained actors to play a particular scenario and tried to make them indiscernible from other service users [24, 29, 30]. This naturalistic method has been used in several previous studies to evaluate various aspects of services delivered by pharmacists [29-33]. Ethical approval was obtained prior to recruitment of pharmacies and simulated smoker-clients from the Queen Mary Research Ethics Committee [Reference: QMREC1830a].

Study Procedure

Recruitment of Pharmacies and STOP Intervention delivery

Our primary aim was to pilot use of the simulated client method as a way to assess fidelity of the refined STOP intervention, with a focus on how well pharmacy staff engage with potential SSS clients. The training therefore focused on communication skills based on motivational interviewing, and practising key phrases such as 'all quit attempts are a success', 'our service is free, delivered with an expert' and 'you can

For peer review only - http://bmjopen.bmj.com/site/about/guidelines.xhtml

come back anytime for support', to facilitate better engagement with potential SSS clients [24].

The STOP team contacted 15 community pharmacies in North East London Boroughs commissioned to deliver the SSS, and recruited six pharmacies. Based on our previous pilot experience and qualitative work, this sample size was deemed appropriate to assess intervention fidelity [24, 16]. One pharmacy subsequently dropped out due to a family emergency. Table 2 outlines the characteristics of the twenty staff members from the five participating pharmacies who individually gave their consent and attended STOP Training. All staff were reimbursed for travel expenses and their time with amounts based on participants' usual day rates. Finally, a WhatsApp group was set up for participating staff as a communication tool and information-sharing platform.

Recruitment of Simulated Clients

Soon after the STOP training was delivered, the STOP team identified and recruited six actors for training to assess pharmacy worker engagement with clients and to record display of smoking cessation materials whilst posing as simulated clients. These simulated clients were purposefully sampled to represent diverse backgrounds reflecting the east London population [34] thus minimising risk of detection by pharmacy staff (Table 3).

Simulated Client Training

All simulated clients attended a 1.5 hour training session led by the STOP team. During the training, each simulated client was assigned one of six smoking-related scenarios (Figure 2) which they practised during the training session and received group feedback. The session also involved demonstrating use of the fidelity assessment questionnaire, developed by the STOP team to assess pharmacy staff smoker engagement behaviour (Figure 3). The fidelity assessment involved noting presence of stop smoking related material in the pharmacy environment, and rating the extent to which pharmacy staff build general rapport and the conversation related to the NHS SSS with the simulated client, using a Likert scale (Figure 3). The questionnaire has a scoring range of 0-36, where higher ratings indicate better client engagement. Items on the questionnaire were chosen to evaluate outputs of the activities represented in the STOP logic model (Figure 1). Simulated clients also provide written feedback on the questionnaire; referred to as field notes, to detail relevant aspects of their interaction where necessary. We used the Qualtrics online survey software [35] enabling simulated clients to complete their fidelity assessments electronically after each visit and aiding immediate receipt of source data for the study team.

The simulated clients practised completing the fidelity assessment questionnaire through Qualtrics; they observed the trainers role-playing interactions between smokers and pharmacy staff, and rated each interaction using Qualtrics. In addition, simulated clients were asked to note the name of each pharmacy worker they interacted with by asking directly or looking at their name badge. If this was not possible, they provided a detailed description of the pharmacy worker instead. This enabled the study team to identify simulated interactions that were with pharmacy staff who had attended the STOP intervention training as not all staff in participating

For peer review only - http://bmjopen.bmj.com/site/about/guidelines.xhtml

pharmacies attended the training. Simulated clients were not aware of the training status of the pharmacy staff they assessed, therefore these were blind outcome assessments.

Fidelity Assessment Pharmacy Visits

Between four and six weeks after STOP intervention training, each simulated client visited every pharmacy once using their simulated smoker-client scenario, resulting in 30 completed fidelity assessments (6 actors x 5 pharmacies). Simulated clients were given two weeks to complete their visits; to facilitate a naturalistic approach no fixed schedule was imposed. All participating pharmacies provided written consent for the visits. However, staff were blind to visit timelines and frequency to minimize risk of detection and resulting change in their usual consulting behaviour. Simulated clients received £30 for every completed fidelity assessment.

Data analysis

Data collected on Qualtrics were analysed using SPSS version 24. Descriptive statistics were used to analyse pharmacy staff recruitment and fidelity assessment outcomes. The simulated clients' field notes were also examined quantitatively, particularly focusing on the number of times smoking cessation materials, and specific actions or phraseology from the STOP training was reported on. Quotes from the field notes were used to exemplify the quantitative data generated by the fidelity assessment tool.

Patient and Public Involvement

No patients and/or public were involved in the development or conduct of this second pilot study. However, we consulted an expert group of pharmacy workers before this study who advised on potential barriers to engaging with STOP intervention.

RESULTS

Smoker engagement ratings

All simulated clients completed their allocated five visits. In total 18 assessments were with pharmacy staff (8 pharmacists or stop smoking advisors, and 10 counter assistants) who had attended the stop training and nine assessments were with pharmacy staff who hand not attended STOP training. On three occasions the training status of the assessed pharmacy worker was unclear.

Table 4 shows total smoker client engagement scores allocated to each pharmacy interaction. Grayscale colours are used to differentiate between interactions with pharmacy staff who attended the STOP intervention training and those who did not. Simulated clients encountered more STOP trained staff in pharmacies with a high staff STOP training attendance. Pharmacies with a higher proportion of trained staff tended to have higher client engagement scores. For example, S06 which was the only pharmacy where all staff attend STOP training was given the highest score by all actors. In contrast, S05 where 40% of its staff attended STOP training had the lowest average client engagement score. Simulated clients rated interactions with STOP

For peer review only

trained staff higher than interactions with staff who did not attend STOP training (Table 5).

Table 6 shows the average ratings each simulated client gave to the five participating pharmacies as part of the fidelity assessment, including display of smoking cessation materials and communication skills. Scores for client rapport indicate that pharmacy staff demonstrated very good or good use of body language and good listening skills. However, use of open questions was limited. In terms of specific smoking-related conversation, pharmacies were given low ratings for indirectly raising the topic of smoking and for highlighting strong evidence for high SSS quit rates. Scores for directly raising the topic of smoking were moderate to low. However, pharmacy staff were rated highly for telling clients about the SSS, the fact that it was heavily subsidised or free for those who do not pay for their prescriptions, and informing clients that they could come back for smoking cessation support at any time.

Display of Stop Smoking Materials

Table 6 shows how many times the simulated clients (n=6) reported seeing specific smoking cessation materials during their five pharmacy visits. The materials were signposted in the STOP training as useful resources for engaging potential smoker-clients to NHS SSS. At least four or more of six simulated clients reported seeing NHS SSS leaflets during their pharmacy visit. Most of the pharmacy staff that the simulated clients encountered did not wear a STOP badge.

Field notes taken by simulated clients

Quantitative analysis of the simulated clients' field notes provided an indication of partial enactment of the STOP Intervention. For example, NHS SSS leaflets were a popular tool for disseminating smoking cessation information and all simulated clients reported being given a leaflet from at least one pharmacy (Table 6). This was also evident in the simulated client comments, with some pharmacies providing customised leaflets, indicating efforts to deliver a more tailored/personalised service.

> *'I was surprised to be given a leaflet with his name printed on it and the times the pharmacy is open and the leaflet did have smoking cessation clinic on it' (actor 6)*
>
> *'Gave out loads of standard (NHS) leaflets plus one he made up himself' (actor 1)*

Aside from leaflets, actors reported being given key details of the NHS SSS proactively in interactions with STOP trained staff. This was at times despite there being other customers in the pharmacy waiting to be served.

> *'Chemist (said) we can help you if you decide to go on the twelve week course with emotional help and medication' (actor 2)*
>
> *'Stressed that I can come anytime for a consultation with himself, it is free, you can just walk in, takes about 15-20 mins gave me leaflet and took the time to go through the 12 week smoking cessation programme despite the pharmacy being busy' (actor 5)*

There was also evidence of clients being offered a smoking cessation appointment by staff at the counter. Simulated clients found most pharmacy staff were proactive in giving the option to book an appointment to see the smoking cessation specialist. A

For peer review only - http://bmjopen.bmj.com/site/about/guidelines.xhtml

few actors were directed to a private room where they spoke with an advisor about joining the NHS SSS.

> *'He did mention that it was a 12 week programme and offered to sign me up a few times' (actor 1)*
>
> *'Very helpful. Offered me the program immediately and nicotine replacement therapy' (actor 6)*
>
> *'Staff at counter were very eager. It was hard to stop them from referring me to their 'trained' person for a private chat.' (actor 4)*

Even when actors declined to join the service, pharmacy staff were keen on giving them smoking cessation leaflets to take home with them or telling clients to come back whenever they felt ready.

> *'Told me about the 12 week program and when I said I had to leave, said I could come back anytime I felt more ready. Got leaflets too' (actor 4)*

These two aspects of giving SSS leaflets and the 'keep the door open' approach were focused on during the training sessions, as several attendees raised the issue of missing potential clients due to the busy pharmacy environment. Throughout group discussions, and role-play exercises, the group felt this approach would particularly help counter staff minimise loss of potential clients needing smoking cessation support.

Several simulated clients described evidence of trained pharmacy staff trying to build rapport by using body language and active listening.

> *'Good eye contact, very pleasant' (actor 2)*
>
> *'Very attentive and listened to content in my scenario' (actor 4)*

On one occasion, one staff member even shared their personal experience of using the service, suggesting a sense of personal commitment to helping others quit.

> *'Self-disclosed that she is using the service and has found it amazing' (actor 6)*

Simulated clients also reported use of key phrases or facts by pharmacy staff that were covered in the STOP training sessions or circulated on the WhatsApp group during their smoking cessation interactions, demonstrating retention of knowledge from the STOP training intervention.

> *'Tom was eager to tell me that my son would need to want this himself … he said there would be little or no point if my son didn't want to stop himself' (actor 6)*
>
> *'70% of smokers want to quit but just need help' (actor 5)*

DISCUSSION

This pilot study suggests that the methods we designed using simulated clients to assess fidelity of a complex intervention worked in practice and gives preliminary evidence of enactment of key intervention components. Findings also indicate that

For peer review only - http://bmjopen.bmj.com/site/about/guidelines.xhtml

client engagement was better for pharmacy staff who attended STOP training, with improved consulting styles and increased use of intervention materials; quantitative analysis of contemporaneous field notes taken by simulated clients confirmed the availability and use of some smoking cessation materials. The analysis also suggested that pharmacy staff (including those without NCSCT training) were using consultation skills and appropriate words and phrases which were taught in their STOP intervention training. From a social cognitive theoretical perspective [36], this potentially demonstrates improved knowledge through vicarious learning and increased self-efficacy to provide basic SSS information to clients.

Strengths

Simulated clients, commonly known as 'mystery shoppers' are widely used in marketing to measure aspects of customer care. As staff are unaware of the simulated client's identity this provides an opportunity for a naturalistic fidelity assessment of how well knowledge and skills from the intervention were received. Similar methods have been found to be rigorous and robust for measuring practice in this setting [29, 31, 32].

The fidelity assessment questionnaire used by the actors allowed for both quantitative and qualitative measurement of pharmacy staff behaviour regarding engagement with smoking cessation services in their working environment. We found that qualitative data from actors' field notes tended to confirm the quantitative ratings.

Our previous research showed that information from the patient coupled with visual and linguistic cues affected advisors' perceptions of the chances of quitting and hence the likelihood of recruitment into the service [15, 16]. Previous studies also confirm that patient characteristics such as age, ethnicity and mental status may form barriers to engaging service users into smoking cessation interventions [15, 37]. This evidence coupled with feedback from our pharmacy staff focus group influenced recruitment of simulated clients from diverse backgrounds presenting a range of different scenarios. Beliefs and attitudes underlying prejudgements of treatment success were addressed in the STOP training [16, 24]. This second pilot study suggests that STOP trained pharmacy staff engaged with all the actors regardless of age and ethnicity and the scenario presented.

The results of this pilot study have informed the STOP logic model in readiness for the STOP trial (Figure 1). For example, site initiation visits and training sessions have been separated because these have a unique purpose and need to take place at different times and in different settings. The outputs in the logic model have been revised to reflect the elements of the intervention which are focussed on staff behaviour, particularly initial client engagement, more clearly.

Weaknesses

Pharmacy staff who chose to attend training may have had different baseline characteristics when compared to those that did not attend. We did not assess engagement skills before the STOP Training intervention to gauge base level of engagement with clients. Nevertheless, our findings are consistent with the suggestion that STOP training may improve pharmacy staff members' ability to engage clients into the NHS SSS. In the main trial we plan to use the same method of fidelity assessment outlined here. However we will include control pharmacies where staff

For peer review only - http://bmjopen.bmj.com/site/about/guidelines.xhtml

have not been offered STOP training which will allow us to compare the performance of pharmacy staff who received STOP training and who did not, to quantify the benefit arising from the intervention.

There was inconsistent reporting of SSS materials seen by simulated clients, particularly in rates of display for posters and the STOP study badge. There were instances where in the same pharmacy some simulated clients reported seeing a poster or leaflets whilst others did not. This may reflect human error, in that there could have been posters up that some simulated clients simply did not see because they were distracted by the busy pharmacy environment. Another explanation could be that since the simulated clients visited at different times, the materials were displayed on some days but not on others.

Strengths and weaknesses in the context of other studies

Previous studies using simulated patients to assess community pharmacy staff performance have focused on provision of over the counter medication [29, 32; 33] whereas our research examines the expanded role of pharmacy staff as agents for health behaviour change. However, we used similar methods: covert visits, blinding to time and number of visits to minimise detection [29, 32]. A key strength of our approach was the use of simulated clients with lived experience of smoking or smoking related health conditions such as asthma. Moreover, feedback from staff in our study indicated no detection of our simulated visits whilst detections were reported in other studies [29, 33].

In this study we assessed the initial interaction between pharmacy worker and smoker which usually takes place over the counter in community pharmacies. However, other researchers have audio-recorded consultations with stop smoking advisors allowing detailed examination of their interactions with clients which take place in a dedicated /private consulting room [8]. Due to ethical considerations related to obtaining patient consent, it was difficult to audio-record naturalistic interactions with pharmacy users within the time limitations of this study. In the main trial, we aim to supplement data from simulated client visits with audiotaped follow-up consultations between stop smoking advisors and actual service users.

One study [30] with a similar aim and methodological approach to ours randomly assigned pharmacies to two different scenarios [38]. With our method, all pharmacies were assessed against all six scenarios thus examining a broader range of skills and allowing a more thorough evaluation of potential gaps in service delivery. In the main trial we will use a balanced design where each pharmacy is exposed to each scenario which will allow more rigorous comparison of the degree to which the intervention is implemented between different pharmacies.

Implications for clinical practice and policy

The methods that we outline here could potentially be adapted to evaluate the effects of any training programme intended to modify the clinical practice of pharmacy staff. Given that government policy in the UK is to expand the range of clinical services provided in pharmacies [39, 40] methods to evaluate the effects of training may be useful in refining interventions and developing new training programmes.

Unanswered questions and future research

For peer review only - http://bmjopen.bmj.com/site/about/guidelines.xhtml

The simulated clients noted adequate display of intervention materials in the pharmacies. However, certain materials were displayed more prominently than others. In the main trial, there is a need to evaluate carefully the use of intervention materials and to understand reasons why certain materials are given more prominence and used more often than others. A better understanding of these factors may lead to development of more effective intervention materials which are more likely to be available to potential users of the intervention.

Author Contributions
SJ led development of the final version of the intervention and designed the fidelity assessment. WYJ and SJ recruited sites, delivered the training, and collected qualitative and quantitative data for this second pilot study. SJ, VM and TY analysed the data. ES led development of the initial intervention for the first pilot study, and gave critical comments on the manuscript alongside WYJ, VM, RS, TY, ST, CG and SE. SJ and RW drafted the paper with input from all other authors. All authors have seen and approved the final manuscript.

Acknowledgments
Mr Darush Attar who is a pharmacist, behaviour change specialist and national public health trainer specialised in smoking cessation, co-facilitated the STOP Intervention training with SJ and WYJ. This work has been previously presented as a thematic poster at the European Respiratory Society Milan 2017 International Congress.

Funding
This is a repilot study of the NIHR-funded STOP programme on which RW is chief investigator and CG, SE and ST are co-investigators. NIHR Programme grant RP-PG-0609-10181.

Competing interests None declared.

Ethics approval Ethical approval for the study was obtained from the Queen Mary Research Ethics Committee [Reference: QMREC1830a]

Provenance and peer review Not commissioned; externally peer reviewed.

Data sharing statement Study data (with identifying information removed) will be available, following completion of the project, on request from the study guarantor, Robert Walton, r.walton@qmul.ac.uk.

For peer review only - http://bmjopen.bmj.com/site/about/guidelines.xhtml

REFERENCES

1. Lancaster T, Stead LF: Individual behavioural counselling for smoking cessation. *Cochrane Database Syst Rev* 2005:CD001292.

2. Stead LF, Lancaster T: Group behaviour therapy programmes for smoking cessation. *Cochrane Database Syst Rev* 2005:CD001007.

3. Stead L, Perera R, Lancaster T: Telephone counseling for smoking cessation. *Cochrane Database Syst Rev* 2006, 2005, CD002850.

4. Michie S, Churchill S, West R: Identifying evidence-based competences required to deliver behavioural support for smoking cessation. *Ann Behav Med* 2011, 41(1):59–70.

5. West R, Stapleton J: Clinical and public health significance of treatments to aid smoking cessation. *Eur Respir Rev* 2008, 17(110):199–204.

6. Lorencatto F, West R, Michie S: Identifying evidence-based behaviour change techniques for smoking cessation in pregnancy. *Nicotine & Tobacco Research* 2012, 14(9):1019–1026. doi:10.1093/ntr/ntr324.

7. Raw M, Regan S, Rigotti NA, McNeill A: A survey of tobacco dependence treatment services in 36 countries. *Addiction* 2009, 104(2):279–87.

8. Lorencatto F, West R, Christopherson C, Michie S. Assessing fidelity of delivery of smoking cessation behavioural support in practice. *Implementation Science*. 2013; 8(1):40.

9. Bauld L, Bell K, McCullough L, Richardson L, Greaves L. The effectiveness of NHS smoking cessation services: a systematic review. Journal of Public Health. 2010 Mar 1;32(1):71-82.

10. Brose LS, West R, Michie S, Kenyon JA, McEwen A. Effectiveness of an online knowledge training and assessment program for stop smoking practitioners. Nicotine & Tobacco Research. 2012 Jan 16;14(7):794-800

11. Anderson C, Blenkinsopp A & Armstrong M, 2009. The contribution of community pharmacy to improving the public's health: summary report of the literature review 1990–2007. Five earlier reports at PharmacyHealthLink.

12. Latif A. & Boardman H. Community pharmacists' attitudes towards medicines use reviews and factors affecting the numbers performed. *Pharm World Sci*, 2008; 30:536–543

13. Saramunee K, Krska J, Mackridge A, Richards J, Suttajit S, Phillips-Howard P. How to enhance public health service utilization in community pharmacy?: General public and health providers' perspectives. Research in Social and Administrative Pharmacy. 2014;10(2):272-84.

For peer review only - http://bmjopen.bmj.com/site/about/guidelines.xhtml

14. Eades CE, Ferguson JS, O'Carroll RE. Public health in community pharmacy: a systematic review of pharmacist and consumer views. BMC public health. 2011;11(1):582.

15. Hudmon KS, Prokhorov AV, Corelli RL. Tobacco cessation counseling: pharmacists' opinions and practices. Patient education and counseling. 2006 Apr 30;61(1):152-60.

16. Sohanpal R, Rivas C, Steed L, MacNeill V, Kuan V, Edwards E, Griffiths C, Eldridge S, Taylor S, Walton R. Understanding recruitment and retention in the NHS community pharmacy stop smoking service: perceptions of smoking cessation advisers. BMJ open. 2016 Jul 1;6(7):e010921.

17. Sinclair H, Bond C, Lennox, AS, et al. An evaluation of a training workshop for pharmacists based on the Stages of Change model of smoking cessation. *Health Education Journal* 1997;56:296-312.

18. Sinclair HK, Bond CM, Lennox AS, et al. Training pharmacists and pharmacy assistants in the stage-of-change model of smoking cessation: a randomised controlled trial in Scotland. *Tob Control* 1998;7(3):253-61.

19. Maguire TA, McElnay JC, Drummond A. A randomized controlled trial of a smoking cessation intervention based in community pharmacies. *Addiction* 2001;96(2):325-31. doi:
10.1080/09652140020021062

20. Greenhalgh T, Macfarlane F, Steed L, Walton R. What works for whom in pharmacist led smoking cessation support: realist review. BMC Medicine 2016 14:209. DOI: 10.1186/s12916-016-0749-5

21. Rivas C, Sohanpal R, McNeill V,Steed L, Edwards E, Griffiths C, Eldridge S, Taylor SJC, Walton R. Defining effective communication strategies in the NHS community pharmacy stop smoking programme: a naturalistic, matched-pairs analysis of smoker consultations. BMJ Open 2017;7:e015664. doi: 10.1136/bmjopen-2016-015664

22. Bock BC, Hudmon KS, Christian J, Graham AL, Bock FR. A tailored intervention to support pharmacy-based counseling for smoking cessation. Nicotine & tobacco research. 2010 Jan 25;12(3):217-25.

23. Madurasinghe VW, Sohanpal R, James W-Y, Steed L, Eldridge SE, Taylor SJC, Griffiths CJ, Walton R. Smoking treatment optimisation in pharmacies (STOP): a cluster randomised pilot trial of a training intervention. Pilot and Feasibility Studies 2017:3:1 DOI: 10.1186/s40814-016-0120-9

24. Steed L, Sohanpal R, James WY, Rivas C, Jumbe S, Chater A, Todd A, Edwards E, MacNeill V, Macfarlane F, Greenhalgh T. Equipping community pharmacy workers as agents for health behaviour change: developing and testing a theory-based intervention. BMJ Open. 2017 Mar 22.

For peer review only - http://bmjopen.bmj.com/site/about/guidelines.xhtml

25. West R, Walia A, Hyder N, et al. Behavior change techniques used by the English Stop Smoking Services and their associations with short-term quit outcomes. *Nicotine Tob Res*
2010;12(7):742-7. doi: 10.1093/ntr/ntq074

26. Breitenstein SM, Gross D, Garvey CA, Hill C, Fogg L, Resnick B. Implementation fidelity in community-based interventions. Research in nursing & health. 2010 Apr 1;33(2):164-73.

27. Boutron I, Moher D, Altman D, Scultz K, Ravaud P: Extending the CONSORT statement to randomized trials of Non-pharmacologic treatment: explanation and elaboration. Annals of Internal Medicine 2008, 148:295–309

28. Rixon L, Baron J, McGale N, Lorencatto F, Francis J, Davies A. Methods used to address fidelity of receipt in health intervention research: a citation analysis and systematic review. BMC health services research. 2016 Dec;16(1):663.

29. Watson MC, Skelton JR, Bond CM, Croft P, Wiskin CM, Grimshaw JM, Mollison J. Simulated patients in the community pharmacy setting–Using simulated patients to measure practice in the community pharmacy setting. Pharmacy World and Science. 2004 Feb 1;26(1):32-7.

30. Saba M, Diep J, Bittoun R, Saini B. Provision of smoking cessation services in Australian community pharmacies: a simulated patient study. International journal of clinical pharmacy. 2014 Jun 1;36(3):604-14.

31. Watson MC, Norris P, Granas AG. A systematic review of the use of simulated patients and pharmacy practice research. International Journal of Pharmacy Practice. 2006 Jun 1;14(2):83-93.

32. Watson MC, Cleland JA, Bond CM. Simulated patient visits with immediate feedback to improve the supply of over-the-counter medicines: a feasibility study. Family practice. 2009 Oct 14;26(6):532-42.

33. Weiss MC, Booth A, Jones B, Ramjeet S, Wong E. Use of simulated patients to assess the clinical and communication skills of community pharmacists. Pharmacy world & science. 2010 Jun 1;32(3):353-61.

34. Robson J, Dostal I, Madurasinghe V, Sheikh A, Hull S, Boomla K, Page H, Griffiths C, Eldridge S. The NHS Health Check programme: implementation in east London 2009–2011. BMJ open. 2015 Apr 1;5(4):e007578.

35. Snow J, Mann M. Qualtrics Survey Software: Handbook for Research Professional. Provo, UT: Qualtrics Labs.

36. Rosvall A, Carlson E. Registered nurses' perception of self-efficacy and competence in smoking cessation after participating in a web-based learning activity. Journal of clinical nursing. 2017 Dec;26(23-24):4777-85.

For peer review only - http://bmjopen.bmj.com/site/about/guidelines.xhtml

37. Williams DM, Newsom JF, Brock TP. An evaluation of smoking cessation-related activities by pharmacists. Journal of the American Pharmaceutical Association (1996). 2000 Mar 1;40(3):366-70.

38. Brose LS, West R, Michie S, et al. Evaluation of Face-to-Face Courses in Behavioural Support for Stop Smoking Practitioners. Journal of Smoking Cessation 2012;7:25-30. doi:doi:10.1017/jsc.2012.6

39. Donovan GR, Paudyal V. England's Healthy Living Pharmacy (HLP) initiative: Facilitating the engagement of pharmacy support staff in public health. Research in social and administrative pharmacy. 2016 Mar 1;12(2):281-92.

40. Twigg MJ, Wright D, Kirkdale CL, Desborough JA, Thornley T. The UK Pharmacy Care Plan service: Description, recruitment and initial views on a new community pharmacy intervention. PloS one. 2017 Apr 3;12(4):e0174500.

For peer review only

Tables

Table 1: Detailed description of the STOP Intervention

For peer review only

PHARMACY SITE INITIATION VISIT	Content	Theoretical Basis [a]	Behaviour change techniques [b]
	Explain the study to pharmacist in charge or manager.<br>Mention potential revenue stream from smoking cessation.<br><br>Emphasise to staff how this fits well with their wider role in health promotion.<br><br>Raise awareness in all staff in preparation for invitation to training.<br><br>Communicate the advantages of the STOP intervention over usual practice, i.e. it is brief and show how it fits with overall 'pharmacy' identity.<br><br>Address pre implementation concerns.<br><br>Provide financial incentive for attending training (only received on completion of training)<br><br>Emphasise backing from local and national opinion leaders and organisations (e.g. Local Pharmaceutical Committee, Royal Pharmaceutical Society, local CCGs and public health commissioners	Adoption by individuals: concerns in preadoption stage (DIT)<br><br>The innovation: compatibility;<br>relative advantage; low complexity (DIT)<br><br>Outer context: incentives (DIT)<br><br>Diffusion and dissemination: opinion leaders (DIT)	10.2 Material reward (behaviour)<br><br>9.1 Credible source<br><br>1.2 Problem Solving<br><br>6.3 Information about others' approval
**TRAINING SESSION**	**Content**	**Theoretical Basis**	**Behaviour change techniques**
**Introduction**	General orientation to STOP programme and aims of training.<br><br>Emphasise backing from local and national opinion leaders and organisations (e.g. Local Pharmaceutical Committee, Royal Pharmaceutical Society, local CCGs and public health commissioners<br><br>Discussion of impact of advisor behaviour on client stop smoking outcomes so far and health benefits to patients from stopping smoking.	Outcome Expectancies (SCT)<br><br>Diffusion and dissemination: opinion leaders (DIT)	5.1 Information on health consequences of behaviour<br><br>9.1 Credible source<br><br>10.6 Nonspecific incentive<br><br>15.1 Verbal persuasion about capability

For peer review only - http://bmjopen.bmj.com/site/about/guidelines.xhtml

	Delivered in mixed groups of pharmacists and other pharmacy workers to promote cohesive working practices within the individual pharmacies.	Implementation and routinisation: organizational structure (DIT)	
**Why are we here?**	Smoking facts and exploration of motivation for helping smokers to quit with feedback.<br><br>Discuss focus on pharmacy setting, emphasising the non-medication related, professional and public health aspects of the pharmacy role.<br><br>Does engaging and supporting smokers' quit fit with role identity, any barriers? Encourage self-perception as supporters and providers of health, how one will feel if help smokers quit.<br><br>Emphasise the non-medication related, professional and public health aspects of the pharmacy role, promote a person-centred rather than product-centred ethos and foster a strong sense of professionalism	Intrinsic and extrinsic motivators (SDT)<br><br>The innovation: compatibility (DIT)	5.6 Information about emotional consequences<br><br>9.2 Pros and cons<br><br>6.3 Information about others approval<br><br>13.1 Identification of self as a role model<br><br>15.3 Focus on past success
**Engaging Clients**	Celebrate successful cases.<br><br>Group exercise and discussion on difficult and easy clients to engage – potential problems and solutions.<br><br>Addressing pharmacy workers beliefs and attitudes e.g. prejudgement of success or failure.	Self-efficacy (SCT)<br>Modelling (SCT)<br>Vicarious learning (SCT)	1.2 Problem solving,<br>8.1 Behavioural practice and rehearsal<br>9.2 Pros and cons<br>13.3 Incompatible beliefs<br>15.3 Focus on past success
**Patient-centred approach: Building rapport & shift of focus**	Introduction of patient centred approach using group exercise. Group identification and discussion of the importance of utilising basic communication skills (rapport, active listening, questioning).<br><br>Review how patient-centred approach can be incorporated into smoking cessation interactions for better patient outcomes<br><br>Role-play demonstration with senior pharmacist, participant practice. How to maximise opportunity with environmental resources e.g. staff wearing STOP badges to prompt client interaction, STOP posters<br><br>Emphasise predictable improved results, simplicity of use and	Self-efficacy (SCT)<br>Modelling (SCT)<br>Vicarious learning (SCT)<br>The innovation: relative advantage; compatibility; low complexity (DIT)	4.1 Instruction on performance of behaviour,<br>6.1 Demonstration of behaviour<br>7.1 Prompts and cues<br>8.1 Behavioural practice and rehearsal<br>8.2 Behaviour substitution<br>8.6 Generalisation of target behaviour<br>9.2 Pros and cons<br>9.3 Comparative imagining of future outcomes

For peer review only - http://bmjopen.bmj.com/site/about/guidelines.xhtml

	benefits over usual practice.		
**NCSCT Knowledge Review**	Review group's NCSCT knowledge with a quiz and general feedback	Self-efficacy (SCT)	1.6 Discrepancy between current behaviour and goal<br>9.1 Credible source
**Pharmacy role in smoking cessation**	Discuss individual pharmacies' NHS Stop Smoking service structure and purpose of smoking treatment, with experienced advisers sharing current and best practice. Group reflection on challenges	Homophily (DIT)	1.7 Review outcome goal(s)
**Behaviour change as smoking cessation treatment**	Emphasise behaviour change support as part of smoking cessation treatment within NHS SSS.<br><br>Information on how to assess someone's readiness to quit smoking using 1-10 scales.	Self-efficacy (SCT)<br>Self-regulation (SCT)<br>The innovation: compatibility (DIT)	4.1 Instruction how to perform behaviour<br>8.1 Behavioural practice and rehearsal
**Behaviour change using patient centred approach in Smoking Cessation Treatment: Double Whammy**	Brainstorm factors that influence behaviour change – (role of beliefs, capability, opportunity alongside knowledge)<br><br>How to elicit individuals' motivations, barriers and potential strategies to change behaviour versus offering solutions. Using 'What else questions'.<br><br>Understanding the 'non-smoker identity' and how to communicate to client<br><br>Demonstration & Role play<br><br>What makes this client centred approach difficult - advantages, disadvantages, barriers and strategies to aid implementation	Outcome Expectancies (SCT)<br>Modelling (SCT)<br>Self-efficacy (SCT)<br>The innovation: fuzzy boundaries (DIT)	1.2 Problem Solving<br>4.1 Instruction on how to perform behaviour<br>6.1 Demonstration of behaviour<br>8.1 Behavioural practice and rehearsal<br>9.2 Pros and Cons
**Client engagement in pharmacy settings: Planning a Quit & Dealing with Lapses**	Discuss planning a quit and how to help people make a specific plan using a SMART approach. Go over ways to discuss with lapses and provide supportive praise.<br><br>Discussion of how to talk about willpower and the role of the open door.<br><br>Watch and reflect on video of strong and weak consultations of quit planning.	Modelling (SCT)<br><br>Self-efficacy (SCT)	1.1 Goal setting (behaviour)<br>1.2 Problem solving<br>3.1 Social support (unspecified)<br>4.1 Instruction on how to perform behaviour<br>6.1 Demonstration of behaviour<br>8.1 Behavioural practice and rehearsal

For peer review only

	Demonstration & Role play		
**Client engagement in pharmacy settings: Goal Setting & Making a Commitment**	Facilitate goal setting and elicit verbal commitment from participants.<br><br>Demonstration (via video)<br><br>Practice cohesive working amongst trainees through role play using multiple scenarios and observer feedback	Modelling (SCT)	1.1 Goal Setting (behaviour)<br>1.9 Commitment<br>3.1 Social support (unspecified)<br>8.1 Behavioural practice and rehearsal<br>15.1 Verbal persuasion about capability
**Implementing STOP**	Review how to implement STOP in practice (i.e. prompts and WhatsApp support) by facilitating discussion of implementation plans alongside facilitators or barriers with pharmacy team<br><br>Highlight use of local champions and prompts/cues including the Double Whammy (a desk calendar with visual cues and example questions to ask) to prompt client interaction.<br><br>Highlight ongoing social support via WhatsApp<br>Promote adaptation of non-core elements of the intervention through a prompted pharmacy team meeting to discuss implementation of the intervention according to the needs of each individual pharmacy e.g. appointment of individual champions, monthly 'STOP' smoking days	Self-regulation (SCT)<br>Intrinsic/Extrinsic Motivators (SDT)<br>The innovation: augmentation/support (DIT)<br>The innovation: trialability; reinvention; fuzzy boundaries; champions (DIT)<br><br>Implementation & routinisation: organisational structure (DIT)	4.1 Instruction on how to perform behaviour<br>1.2 Problem Solving<br>7.1 Prompts and Cues<br>3.2 Social support (practical)<br>1.4 Action planning<br><br>10.1 Material incentive (behaviour)
**End of Session**	Participants provided with a certificate for attending the training linked to CPD (endorsed by RPS)<br><br>Provide financial reward for those who have completed intervention training	Outer context: incentives (DIT)	10.2 Material reward
**6 WEEK BOOSTER VISIT**	**Content**	**Theoretical Basis**	**Behaviour change techniques**
**Feedback on smoker engagement and update on STOP implementation**	Identification of organizational barriers, facilitators to implementing STOP in individual pharmacies. Facilitating action plans to implement STOP in their pharmacy.<br><br>Any further thoughts on how the intervention can be adapted to local circumstances?<br><br>Review pharmacy staffs' self-efficacy of skills	The innovation: fuzzy boundaries (DIT)<br>Adoption by individuals: concerns in preadoption stage (DIT)<br><br>The innovation: reinvention	1.1 Goal Setting<br>1.2 Problem solving<br>1.4 Action Planning<br>1.5 Review behaviour goal(s)<br>1.6 Discrepancy between current behaviour and goal<br>1.7 Review outcome goal<br>1.9 Commitment

For peer review only

	Troubleshoot based on performance feedback, assessment by simulated clients* and staff's self-reported self-efficacy<br><br>*Simulated clients are trained actors who approach staff in intervention pharmacies using smoking-related scenarios to assess client engagement and evaluate presence of STOP smoking environmental cues (posters, badges)	The innovation: augmentation/support (DIT)<br><br>Self-efficacy (SCT)<br><br>The innovation: trialability (DIT)	2.2 Feedback on behaviour<br>2.7 Feedback on outcome of behaviour<br><br>7.1 Prompts and cues<br><br>8.3 Habit formation

Footnote
[a] DIT refers to 'Diffusion of Innovation' Theory. See Greenhalgh T, et al. Diffusion of innovations in service organizations: systematic review and recommendations. The Milbank Quarterly. 2004 Dec;82(4):581-629.
SCT refers to 'Social Cognitive Theory'. See Bandura A. Health promotion from the perspective of social cognitive theory. Psychology and health. 1998 Jul 1;13(4):623-49.
[b] derived from the Behaviour Change taxonomy (Michie S. et al The behavior change technique taxonomy (v1) of 93 hierarchically clustered techniques: building an international consensus for the reporting of behavior change interventions. Annals of behavioral medicine. 2013 Mar 20;46(1):81-95.

For peer review only

Table 2: Pharmacy Staff Demographics

CHARACTERISTICS		SUPPORT STAFF (n=16)	STOP SMOKING ADVISORS (n=4)	Total
Mean age in years (Range)		29 (16-49)	37 (30-54)	30 (16-54)
Male (%)		38	100	50
Graduate or higher (%)		25	100	40
Never smoked (%)		75	100	80
Job titles	**Counter Assistant**	10		
	**Dispensing Chemist**	4		
	**Trainee pharmacist**	2		
	**Pharmacist**		2	
	**Pharmacist technician**		1	
	**Business Manager**		1	

Table 3: Simulated clients demographics

ID	Age	Gender	Ethnicity	Education	Smoking Status
01	49	Male	White	Postgraduate	Never smoked
02	56	Female	White British	Other	Ex-smoker
03	54	Male	Mixed	Graduate	Ex-smoker
04	32	Male	Black	Graduate	Never smoked
05	22	Female	Mixed	NVQ L3	Ex-smoker
06	58	Female	White British	Professional (CPCAB)	Ex-smoker

For peer review only - http://bmjopen.bmj.com/site/about/guidelines.xhtml

Table 4: Client engagement ratings from simulated smokers

		Client Engagement Ratings by simulated clients						
		**1**	**2**	**3**	**4**	**5**	**6**	***Average score***
S06	**100**	36	34	36	34	34	29	35
S02	**60**	29	14	18	29	6	14	18
S01	**57**	18	14	16	25	27	6	18
S05	**40**	10	4	20	15	28	14	15
S03	**33**	14	24	25	27	22	20	22
PHARMACY SITE	***% of pharmacy staff trained***	**LEGEND**<br>☐ Completed STOP training<br>■ Did not attend training<br>▒ Unable to identify						

For peer review only - http://bmjopen.bmj.com/site/about/guidelines.xhtml

Table 5: Mean Client Engagement Scores for trained *vs* untrained staff

	ATTENDED STOP TRAINING	N	Mean	Std. Dev.
OVERALL CLIENT ENGAGEMENT	No	12	16.9	7.7
	Yes	18	24.4	9.0
BUILDING RAPPORT	No	12	6.7	3.7
	Yes	18	9.1	2.3
CONVERSATION	No	12	10.3	5.1
	Yes	18	15.3	7.1

For peer review only

Table 6: Average simulated client fidelity assessment ratings including display of smoking cessation materials and communication skills

	SC 1	SC 2	SC 3	SC 4	SC 5	SC 6
**Display of Smoking Cessation Materials**						
NHS SSS Poster / audio information	Y= 3	Y= 1	Y= 0	Y= 5	Y= 0	Y= 2
NHS SSS Leaflets	Y= 4	Y= 5	Y= 3	Y= 5	Y= 5	Y= 5
STOP study poster	Y= 1	Y= 1	Y= 2	Y= 3	Y= 0	Y= 3
STOP study badge	Y= 0	Y= 1	Y= 1	Y= 1	Y= 0	Y= 1
***Rapport with clients**						
Good use of body language	2	2	2	3	3	2
Good listening skills	2	2	2	3	3	2
Use of open questions	1	1	2	1	2	2
Picking up client's verbal or visual cues	2	2	2	2	3	2
***Conversation**						
Initiate conversation on smoking in response to cues	2	1	2	3	2	2
Raise smoking directly	2	1	1	2	2	0
Raise smoking indirectly	1	0	1	1	0	0
Tell client about available SSS in pharmacy	2	3	3	3	2	2
Highlight free or subsidised service	2	3	3	2	2	1
Highlight facts on SSS high quit rates	1	1	1	1	1	1
Ask client about service referral	2	1	3	2	2	1
Close conversation with 'come back anytime' for help	2	2	2	3	2	2

Key
SC = simulated client
'Y=' refers to the total number of times a simulated client ticked to confirm display of a smoking cessation material from their 5 pharmacy visits
* Numbers here refer to average client engagement scores assigned by simulated smokers across their 5 pharmacy visits. Range of 0-3, where 0 indicates no rapport or conversation and 3 indicates very good rapport or conversation

For peer review only

Figures

Figure 1: STOP Programme Final Logic Model

Figure 2: Smoking-related scenarios

Figure 3: Fidelity Assessment Questionnaire

For peer review only

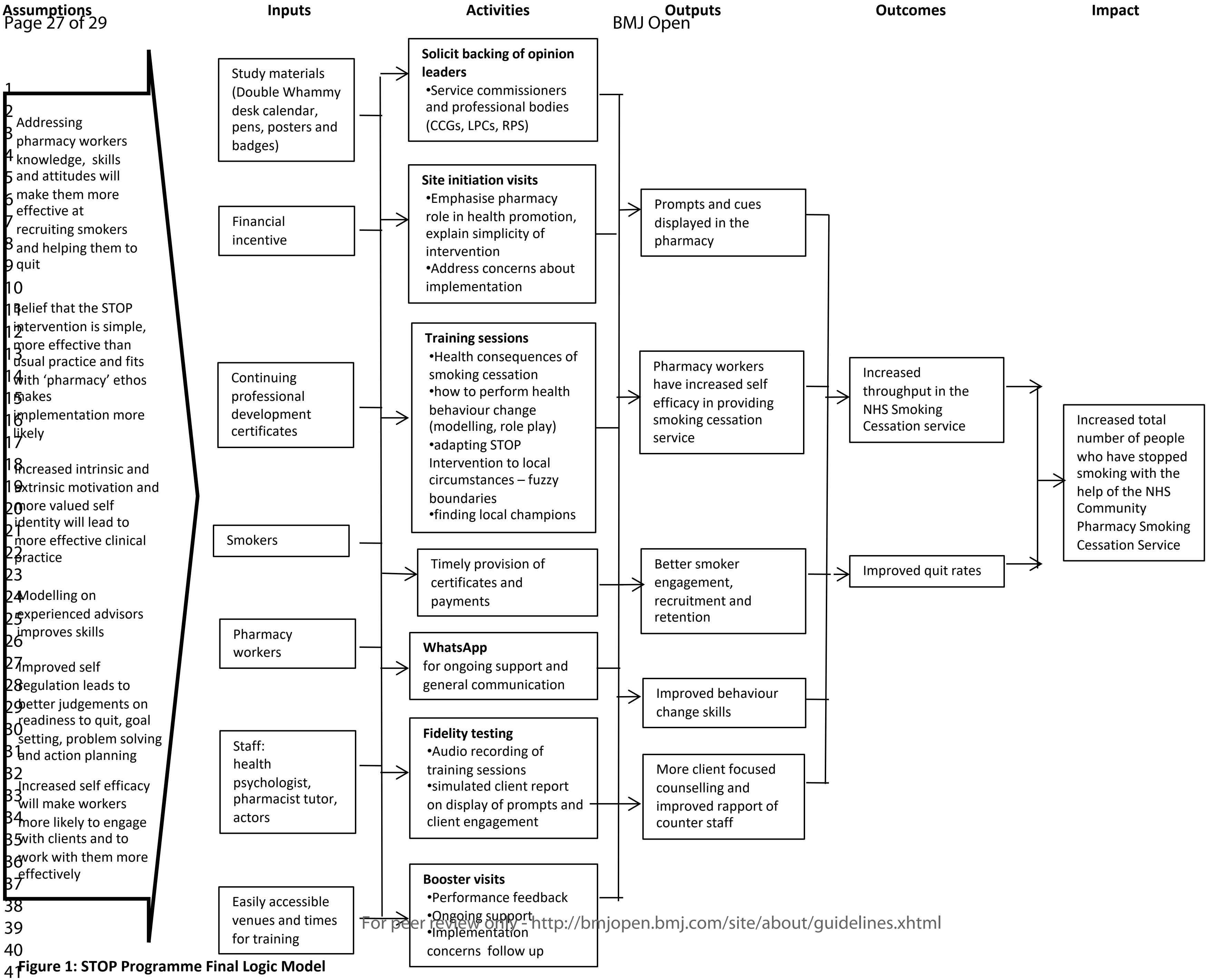

For peer review only - http://bmjopen.bmj.com/site/about/guidelines.xhtml

Figure 1: STOP Programme Final Logic Model

Figure 2: Brief scenario outlines

**Scenario 1**<br>Individual picks up SSS visual resource and reads through, attends counter and asks if the pharmacy offers advice on how to stop smoking. Has discovered teenage child smokes and wants them to stop.<br><br>Actor required: middle aged woman, white	**Scenario 2**<br>Individual attends counter coughing, with cigarettes obviously on display e.g. holding in hand and putting on counter. Individual tells pharmacy staff they have asthma and need advice on how to stop smoking.<br><br>Actor required: middle aged male, white	**Scenario 3**<br>Individual attends counter with non-smoking related request e.g. purchase pregnancy vitamins. Has cigarettes visible and obviously on display e.g. holding in hand and putting on counter.<br><br>Actor required: middle aged male, ethnic minority
**Scenario 4**<br>Individual attends counter wheezing, with asthma pump e.g. holding in hand and putting on counter. Individual tells pharmacy staff they have asthma and COPD but still smoke. Asthma is getting worse so they need advice on how to stop smoking.<br><br>Actor required: middle aged female, white	**Scenario 5**<br>Individual attends the pharmacy counter, complaining of a sore throat and need of lozenges. Explains that they went partying last night with friends and was smoking so maybe sore throat is linked to that. Friend and mother nagging that sore throat is due to smoking. Ask pharmacy worker what they think it could be?<br><br>Actor required: young female, ethnic minority	**Scenario 6**<br>Individual attends counter, complaining of stress from girlfriend/mum to stop smoking because he has been getting sore throats a lot lately. Not sure if he wants to quit but thought he should check out what the pharmacy have to offer. Saw a sign that says pharmacy has a Stop Smoking Service.<br><br>Actor required: young male, ethnic minority

For peer review only

Actor ID: ___ ___

Fidelity Assessment Questionnaire

Date:<br>DD / MM / YEAR	Time:<br>__ : __ hours	Scenario number:	Pharmacy ID

Environment

Were the following visible within the pharmacy:-	***Tick if seen***
NHS Stop Smoking Service poster or audiovisual information	
NHS Stop Smoking Service leaflets	
STOP Study Poster	
Staff wearing STOP Study Badges	

Rapport

To what extent did the pharmacy worker demonstrate:- (circle a number)

a) good body language e.g. eye contact, attentiveness 0 1 2 3
b) good listening skills e.g. focused on conversation 0 1 2 3
c) use of open questions 0 1 2 3
d) picking up on client's verbal or visual cues 0 1 2 3

Conversation

To what extent did the pharmacy worker:- (circle a number)

a) Initiate conversation of smoking in response to a verbal or visual prompt 0 1 2 3
b) Raise smoking directly e.g. do you smoke 0 1 2 3
c) Raise smoking indirectly e.g. by talking about second hand smoke 0 1 2 3
d) Tell you there is a smoking cessation service available in the pharmacy 0 1 2 3
e) Highlight that the service is free aside from NRT if pay for prescriptions 0 1 2 3
f) Highlight that people who use the service are 4 times more likely to quit 0 1 2 3
h) Ask you whether you want a referral to the service 0 1 2 3
i) Close conversation by saying you can come back anytime for help/support 0 1 2 3

Additional comments:	Total score:

For peer review only

BMJ Open

Evaluating NHS stop smoking service engagement in community pharmacies using simulated smokers: fidelity assessment of a theory-based intervention

Journal:	*BMJ Open*
Manuscript ID	bmjopen-2018-026841.R2
Article Type:	Research
Date Submitted by the Author:	18-Feb-2019
Complete List of Authors:	Jumbe, Sandra; Barts and The London School of Medicine and Dentistry, Centre for Primary Care and Public Health; Queen Mary University of London<br>James, Wai Yee; Barts and The London School of Medicine and Dentistry, Centre for Primary Care and Public Health<br>Madurasinghe, Vichithranie; Barts and The London School of Medicine and Dentistry,<br>Steed, Liz; Barts and The London School of Medicine and Dentistry, Centre for Primary Care and Public Health<br>Sohanpal, Ratna; Queen Mary University of London, Centre for Primary Care and Public Health<br>Yau, Tammy; California Northstate University, Medicine<br>Taylor, Stephanie; Queen Mary University of London, Centre for Primary Care and Public Health<br>Eldridge, Sandra; Queen Mary University of London, Centre for Primary Care and Public Health<br>Griffiths, Chris; Barts and The London School of Medicine and Dentistry,<br>Walton, Robert; Barts and The London School of Medicine and Dentistry, Centre for Health Sciences
**Primary Subject Heading**:	Smoking and tobacco
Secondary Subject Heading:	Health services research, Public health, Research methods
Keywords:	Smoking cessation, community pharmacies, fidelity assessment, simulated patients

SCHOLARONE™
Manuscripts

Article Title: Evaluating NHS stop smoking service engagement in community pharmacies using simulated smokers: fidelity assessment of a theory-based intervention

Sandra Jumbe[1]

Wai Yee James[1]

Vichithranie Madurasinghe[1]

Liz Steed[1]

Ratna Sohanpal[1]

Tammy Yau[2]

Stephanie JC Taylor[1]

Sandra Eldridge[1]

Chris Griffiths[1]

Robert Walton[1]

[1] Barts and The London School of Medicine and Dentistry, Centre for Primary Care and Public Health, Queen Mary University of London, 58 Turner Street, London E1 2AB. United Kingdom
[2] California Northstate University, 9700 West Taron Drive, Elk Grove, CA 95757 USA

Corresponding author:
Dr Sandra Jumbe
Centre for Primary Care and Public Health
Queen Mary University of London
58 Turner Street
London
E1 2AB
Phone: +442078823448
Fax: +442078822552
Email: s.jumbe@qmul.ac.uk

Keywords: smoking cessation, pharmacies, simulated smokers, fidelity assessment

Word count: 3890

For peer review only

ABSTRACT

Objectives: Smokers are more likely to quit if they use the NHS Stop Smoking Service (SSS). However, community pharmacies experience low service uptake. The Smoking Treatment Optimisation in Pharmacies (STOP) programme aims to address this problem by enhancing staff training using a theory-based intervention. In this study we evaluated intervention fidelity using simulated smokers (actors) to assess smoker engagement and enactment of key intervention components by STOP trained staff.

Design: Observational pilot study.

Settings: Five community pharmacies in North East London with an NHS SSS

Methods: Six actors, representative of East London's population, were recruited and trained to complete intervention fidelity assessments. Consenting pharmacy staff from five participating pharmacies received STOP Intervention training. Four weeks after the staff training, the actors visited the participating pharmacies posing as smokers eligible for smoking cessation support. Engagement behaviour by pharmacy staff and enactment of intervention components was assessed using a scoring tool derived from the STOP logic model (scoring range of 0-36), and contemporaneous field notes taken by actors.

Results: 18 of 30 completed assessments were with STOP trained staff (10/18 were counter assistants). Mean score for smoker engagement was 24.4, (SD 9.0) points for trained and 16.9 (SD 7.8) for untrained staff, respectively. NHS SSS leaflets (27/30) were the most common smoking cessation materials seen on pharmacy visits. Most trained counter staff engaged with smokers using leaflets and a few proactively offered appointments with their cessation advisors. Appropriate use of body language was reported on 26/30 occasions alongside use of key phrases from the STOP training session (n=8). Very few pharmacy staff wore STOP promotional badges (4/30).

Conclusions: STOP training may change client engagement behaviour in pharmacy staff and could improve uptake of the NHS SSS. A cluster randomised controlled trial is currently in progress to evaluate its effectiveness and cost effectiveness.

Article Summary

Strengths and limitations of the study

- We used simulated clients for a naturalistic fidelity assessment measuring enactment of a complex intervention to promote use of smoking cessation services in community pharmacies
- The method enables quantitative and qualitative evaluation of pharmacy staff behaviour regarding client engagement by assessing whether key intervention materials are made available to service users, and rating what staff say and do.
- We found that this method worked well and gave an indication that important elements of the intervention were being enacted in the pharmacies, however lack of comparison data means we cannot necessarily attribute these findings to the STOP intervention training.
- This study shows the simulated client assessment method is feasible, and we will use this in the main trial to compare intervention and control pharmacies as part of the process evaluation.

For peer review only

BACKGROUND

Behavioural support for smoking cessation is highly cost-effective in reducing tobacco related morbidity and mortality [1-3]. Behavioural interventions include advice, discussion, and targeted activities aiming to: minimise motivation to smoke; increase resolve not to smoke; facilitate strategies to reduce exposure to smoking cues; improve management of smoking urges; promote smoking cessation medication [4-6]. Rising prominence of evidence-based practice has resulted in increased implementation of behavioural support interventions as part of routine healthcare [7]. One example of this is the NHS Stop Smoking Service (SSS) in the United Kingdom (UK), which offers smoking cessation treatment including nicotine replacement therapy (NRT) to smokers trying to quit, alongside weekly consultations [8]. Results indicate that smokers engaged with this service are four times more likely to quit than those using NRT alone [9].

Pharmacies with at least one staff member who has completed National Centre for Smoking Cessation and Training (NCSCT) training, often known as a stop smoking advisor (SSA), are able to deliver the NHS SSS [10]. There is strong evidence for the success of the pharmacy-led SSS in cost-effectiveness and good abstinence rates, endorsing behaviour change training of community pharmacy staff as an effective way of helping people to stop smoking [11]. However, service uptake in pharmacy settings is low [12]. Whilst the recent decrease in smoking prevalence in the UK may be a factor, low uptake may also arise from lack of awareness of pharmacies' public health role [13]. Studies also suggest low pharmacy staff confidence in their ability to deliver such services linked to expectation of negative reactions from customers [13-16].

Previous studies looking at the impact of smoking cessation training for pharmacists suggest a range of benefits including increased levels of counselling [17, 18], improved pharmacist consulting behaviour [19-21], and higher quit rates [19, 22]. Whilst previous interventions have shown benefits, their focus was primarily on the smoking cessation consultation itself rather than initial smoker engagement. The Smoking Treatment Optimisation in Pharmacies (STOP) programme was established to enhance delivery of the NHS SSS [23] by targeting self-efficacy, motivation of pharmacy workers and skills to increase smoker engagement [24].

This theory-based complex intervention was developed and tested for acceptability in twelve community pharmacies from three east London boroughs in an initial pilot study [24]. Specifically, 20 SSAs from these pharmacies attended two skills-based training sessions focused on communication and behaviour change skills. Study results confirmed the acceptability of the STOP intervention in terms of overall structure and face-to-face training content, with some participants reporting use of newly learnt skills in practice. However, organisational barriers such as limited finances to cover pharmacist absence and poor acceptability of training venue and times limited pharmacy staff attendance. Another key finding was that very few staff members working at the pharmacy counter attended STOP training sessions. This limited effective service delivery because they were less able to engage in smoking-related conversation with clients [24].

To address logistical barriers highlighted in the first pilot study for attending training, the study team conducted a focus group with four pharmacy-based SSAs and two

For peer review only - http://bmjopen.bmj.com/site/about/guidelines.xhtml

counter staff. Based on feedback from this group several changes were made to the intervention to enhance attendance and intervention uptake. Firstly, training delivery was changed from two sessions to a half-day session on a Sunday morning with an option for onsite training on a weekday if more convenient to the pharmacy owner. Secondly, we took an organisational approach training all pharmacy staff (both NCSCT trained and untrained) within the same session to facilitate shared responsibility and focus on initial client engagement. The refined STOP Training Intervention content is summarised in Table 1 which outlines the theories and behaviour change techniques [24, 25] upon which the intervention is based. The STOP logic model describing the programme theory for the intervention is shown in Figure 1.

To address receipt of the refined STOP intervention, we conducted a second pilot in November 2016. The focus of this second pilot study was to assess the fidelity of the refined STOP intervention, that is, to evaluate pharmacy staff performance of intervention skills in practice. Fidelity of intervention delivery involves assessing the extent to which core, prescribed intervention components are delivered as intended and are received by participants [26]. These two elements are critical for successful translation of evidence-based interventions into practice [26, 27]. Results from fidelity assessments may highlight how the intervention is working and aspects that could be improved. However, fidelity assessment methods are not frequently reported [26-28]. In this study we piloted the use of the 'simulated client' method as a way of assessing fidelity of the refined STOP intervention, with a focus on how well pharmacy staff engage with potential SSS clients [29-30]. Specifically, this paper reports on the potential of this method, to measure enactment of key elements of the intervention and evaluate impact of STOP training on client engagement into the SSS by pharmacy staff.

METHODS

Study Design

This was an observational pilot study conducted in North East London Boroughs where simulated clients were used to examine how community pharmacies engage with pharmacy users asking for smoking cessation advice. We trained actors to play a particular scenario and tried to make them indiscernible from other service users [24, 29, 30]. This naturalistic method has been used in several previous studies to evaluate various aspects of services delivered by pharmacists [29-33]. Ethical approval was obtained prior to recruitment of pharmacies and simulated smoker-clients from the Queen Mary Research Ethics Committee [Reference: QMREC1830a].

Study Procedure

Recruitment of Pharmacies and STOP Intervention delivery

Our primary aim was to pilot use of the simulated client method as a way to assess fidelity of the refined STOP intervention, with a focus on how well pharmacy staff engage with potential SSS clients. The training therefore focused on communication skills based on motivational interviewing and practising key phrases such as 'all quit attempts are a success', 'our service is free, delivered with an expert' and 'you can

For peer review only - http://bmjopen.bmj.com/site/about/guidelines.xhtml

come back anytime for support', to facilitate better engagement with potential SSS clients [24].

The STOP team contacted 15 community pharmacies in North East London Boroughs commissioned to deliver the SSS, and recruited six pharmacies. Based on our previous pilot experience and qualitative work, this sample size was deemed appropriate to assess intervention fidelity [24, 16]. One pharmacy subsequently dropped out due to a family emergency. Table 2 outlines the characteristics of the twenty staff members from the five participating pharmacies who individually gave their consent and attended STOP Training. All staff were reimbursed for travel expenses and their time with amounts based on participants' usual day rates. Finally, a WhatsApp group was set up for participating staff as a communication tool and information-sharing platform.

Recruitment of Simulated Clients

Soon after the STOP training was delivered, the STOP team identified and recruited six actors for training to assess pharmacy worker engagement with clients and to record display of smoking cessation materials whilst posing as simulated clients. These simulated clients were purposefully sampled to represent diverse backgrounds reflecting the east London population [34] thus minimising risk of detection by pharmacy staff (Table 3).

Simulated Client Training

All simulated clients attended a 1.5 hour training session led by the STOP team. During the training, each simulated client was assigned one of six smoking-related scenarios (Figure 2) which they practised during the training session and received group feedback. The session also involved demonstrating use of the fidelity assessment questionnaire, developed by the STOP team to assess pharmacy staff smoker engagement behaviour (Figure 3). The fidelity assessment involved noting presence of stop smoking related material in the pharmacy environment, and rating the extent to which pharmacy staff build general rapport and the conversation related to the NHS SSS with the simulated client, using a Likert scale (Figure 3). The questionnaire has a scoring range of 0-36, where higher ratings indicate better client engagement. Items on the questionnaire were chosen to evaluate outputs of the activities represented in the STOP logic model (Figure 1). Simulated clients also provide written feedback on the questionnaire; referred to as field notes, to detail relevant aspects of their interaction where necessary. We used the Qualtrics online survey software [35] enabling simulated clients to complete their fidelity assessments electronically after each visit and aiding immediate receipt of source data for the study team.

The simulated clients practised completing the fidelity assessment questionnaire through Qualtrics; they observed the trainers role-playing interactions between smokers and pharmacy staff, and rated each interaction using Qualtrics. In addition, simulated clients were asked to note the name of each pharmacy worker they interacted with by asking directly or looking at their name badge. If this was not possible, they provided a detailed description of the pharmacy worker instead. This enabled the study team to identify simulated interactions that were with pharmacy staff who had attended the STOP intervention training as not all staff in participating

For peer review only - http://bmjopen.bmj.com/site/about/guidelines.xhtml

pharmacies attended the training. Simulated clients were not aware of the training status of the pharmacy staff they assessed, therefore these were blind outcome assessments.

Fidelity Assessment Pharmacy Visits

Between four and six weeks after STOP intervention training, each simulated client visited every pharmacy once using their simulated smoker-client scenario, resulting in 30 completed fidelity assessments (6 actors x 5 pharmacies). Simulated clients were given two weeks to complete their visits; to facilitate a naturalistic approach no fixed schedule was imposed. All participating pharmacies provided written consent for the visits. However, staff were blind to visit timelines and frequency to minimize risk of detection and resulting change in their usual consulting behaviour. Simulated clients received £30 for every completed fidelity assessment.

Data analysis

Data collected on Qualtrics were analysed using SPSS version 24. Descriptive statistics were used to analyse pharmacy staff recruitment and fidelity assessment outcomes. The simulated clients' field notes were also examined quantitatively, particularly focusing on the number of times smoking cessation materials, and specific actions or phraseology from the STOP training was reported on. Quotes from the field notes were used to exemplify the quantitative data generated by the fidelity assessment tool.

Patient and Public Involvement

No patients and/or public were involved in the development or conduct of this second pilot study. However, we consulted an expert group of pharmacy workers before this study who advised on potential barriers to engaging with STOP intervention.

RESULTS

Smoker engagement ratings

All simulated clients completed their allocated five visits. In total 18 assessments were with pharmacy staff (8 pharmacists or stop smoking advisors, and 10 counter assistants) who had attended the stop training and nine assessments were with pharmacy staff who hand not attended STOP training. On three occasions the training status of the assessed pharmacy worker was unclear.

Table 4 shows total smoker client engagement scores allocated to each pharmacy interaction. Grayscale colours are used to differentiate between interactions with pharmacy staff who attended the STOP intervention training and those who did not. Simulated clients encountered more STOP trained staff in pharmacies with a high staff STOP training attendance. Pharmacies with a higher proportion of trained staff tended to have higher client engagement scores. For example, S06 which was the only pharmacy where all staff attend STOP training was given the highest score by all actors. In contrast, S05 where 40% of its staff attended STOP training had the lowest average client engagement score. Simulated clients rated interactions with STOP

For peer review only

trained staff higher than interactions with staff who did not attend STOP training (Table 5).

Table 6 shows the average ratings each simulated client gave to the five participating pharmacies as part of the fidelity assessment, including display of smoking cessation materials and communication skills. Scores for client rapport indicate that pharmacy staff demonstrated very good or good use of body language and good listening skills. However, use of open questions was limited. In terms of specific smoking-related conversation, pharmacies were given low ratings for indirectly raising the topic of smoking and for highlighting strong evidence for high SSS quit rates. Scores for directly raising the topic of smoking were moderate to low. However, pharmacy staff were rated highly for telling clients about the SSS, the fact that it was heavily subsidised or free for those who do not pay for their prescriptions, and informing clients that they could come back for smoking cessation support at any time.

Display of Stop Smoking Materials

Table 6 shows how many times the simulated clients (n=6) reported seeing specific smoking cessation materials during their five pharmacy visits. The materials were signposted in the STOP training as useful resources for engaging potential smoker-clients to NHS SSS. At least four or more of six simulated clients reported seeing NHS SSS leaflets during their pharmacy visit. Most of the pharmacy staff that the simulated clients encountered did not wear a STOP badge.

Field notes taken by simulated clients

Quantitative analysis of the simulated clients' field notes provided an indication of partial enactment of the STOP Intervention. For example, NHS SSS leaflets were a popular tool for disseminating smoking cessation information and all simulated clients reported being given a leaflet from at least one pharmacy (Table 6). This was also evident in the simulated client comments, with some pharmacies providing customised leaflets, indicating efforts to deliver a more tailored/personalised service.

> *'I was surprised to be given a leaflet with his name printed on it and the times the pharmacy is open and the leaflet did have smoking cessation clinic on it' (actor 6)*
>
> *'Gave out loads of standard (NHS) leaflets plus one he made up himself' (actor 1)*

Aside from leaflets, actors reported being given key details of the NHS SSS proactively in interactions with STOP trained staff. This was at times despite there being other customers in the pharmacy waiting to be served.

> *'Chemist (said) we can help you if you decide to go on the twelve week course with emotional help and medication' (actor 2)*
>
> *'Stressed that I can come anytime for a consultation with himself, it is free, you can just walk in, takes about 15-20 mins gave me leaflet and took the time to go through the 12 week smoking cessation programme despite the pharmacy being busy' (actor 5)*

There was also evidence of clients being offered a smoking cessation appointment by staff at the counter. Simulated clients found most pharmacy staff were proactive in giving the option to book an appointment to see the smoking cessation specialist. A

For peer review only - http://bmjopen.bmj.com/site/about/guidelines.xhtml

few actors were directed to a private room where they spoke with an advisor about joining the NHS SSS.

> *'He did mention that it was a 12 week programme and offered to sign me up a few times' (actor 1)*
>
> *'Very helpful. Offered me the program immediately and nicotine replacement therapy' (actor 6)*
>
> *'Staff at counter were very eager. It was hard to stop them from referring me to their 'trained' person for a private chat.' (actor 4)*

Even when actors declined to join the service, pharmacy staff were keen on giving them smoking cessation leaflets to take home with them or telling clients to come back whenever they felt ready.

> *'Told me about the 12 week program and when I said I had to leave, said I could come back anytime I felt more ready. Got leaflets too' (actor 4)*

These two aspects of giving SSS leaflets and the 'keep the door open' approach were focused on during the training sessions, as several attendees raised the issue of missing potential clients due to the busy pharmacy environment. Throughout group discussions, and role-play exercises, the group felt this approach would particularly help counter staff minimise loss of potential clients needing smoking cessation support.

Several simulated clients described evidence of trained pharmacy staff trying to build rapport by using body language and active listening.

> *'Good eye contact, very pleasant' (actor 2)*
>
> *'Very attentive and listened to content in my scenario' (actor 4)*

On one occasion, one staff member even shared their personal experience of using the service, suggesting a sense of personal commitment to helping others quit.

> *'Self-disclosed that she is using the service and has found it amazing' (actor 6)*

Simulated clients also reported use of key phrases or facts by pharmacy staff that were covered in the STOP training sessions or circulated on the WhatsApp group during their smoking cessation interactions, demonstrating retention of knowledge from the STOP training intervention.

> *'Tom was eager to tell me that my son would need to want this himself … he said there would be little or no point if my son didn't want to stop himself' (actor 6)*
>
> *'70% of smokers want to quit but just need help' (actor 5)*

DISCUSSION

This pilot study suggests that the methods we designed using simulated clients to assess fidelity of a complex intervention worked in practice and gives preliminary evidence of enactment of key intervention components. Findings also indicate that

For peer review only - http://bmjopen.bmj.com/site/about/guidelines.xhtml

client engagement was better for pharmacy staff who attended STOP training, with improved consulting styles and increased use of intervention materials; quantitative analysis of contemporaneous field notes taken by simulated clients confirmed the availability and use of some smoking cessation materials. The analysis also suggested that pharmacy staff (including those without NCSCT training) were using consultation skills and appropriate words and phrases which were taught in their STOP intervention training. From a social cognitive theoretical perspective [36], this potentially demonstrates improved knowledge through vicarious learning and increased self-efficacy to provide basic SSS information to clients.

Strengths

Simulated clients, commonly known as 'mystery shoppers' are widely used in marketing to measure aspects of customer care. As staff are unaware of the simulated client's identity this provides an opportunity for a naturalistic fidelity assessment of how well knowledge and skills from the intervention were received. Similar methods have been found to be rigorous and robust for measuring practice in this setting [29, 31, 32].

The fidelity assessment questionnaire used by the actors allowed for both quantitative and qualitative measurement of pharmacy staff behaviour regarding engagement with smoking cessation services in their working environment. We found that qualitative data from actors' field notes tended to confirm the quantitative ratings.

Our previous research showed that information from the patient coupled with visual and linguistic cues affected advisors' perceptions of the chances of quitting and hence the likelihood of recruitment into the service [15, 16]. Previous studies also confirm that patient characteristics such as age, ethnicity and mental status may form barriers to engaging service users into smoking cessation interventions [15, 37]. This evidence coupled with feedback from our pharmacy staff focus group influenced recruitment of simulated clients from diverse backgrounds presenting a range of different scenarios. Beliefs and attitudes underlying prejudgements of treatment success were addressed in the STOP training [16, 24]. This second pilot study suggests that STOP trained pharmacy staff engaged with all the actors regardless of age and ethnicity and the scenario presented.

The results of this pilot study have informed the STOP logic model in readiness for the STOP trial (Figure 1). For example, site initiation visits and training sessions have been separated because these have a unique purpose and need to take place at different times and in different settings. The outputs in the logic model have been revised to reflect the elements of the intervention which are focussed on staff behaviour, particularly initial client engagement, more clearly.

Weaknesses

Pharmacy staff who chose to attend training may have had different baseline characteristics when compared to those that did not attend. We did not assess engagement skills before the STOP Training intervention to gauge base level of engagement with clients. Nevertheless, our findings are consistent with the suggestion that STOP training may improve pharmacy staff members' ability to engage clients into the NHS SSS. In the main trial we plan to use the same method of fidelity assessment outlined here. However we will include control pharmacies where staff

For peer review only - http://bmjopen.bmj.com/site/about/guidelines.xhtml

have not been offered STOP training which will allow us to compare the performance of pharmacy staff who received STOP training and who did not, to quantify the benefit arising from the intervention.

There was inconsistent reporting of SSS materials seen by simulated clients, particularly in rates of display for posters and the STOP study badge. There were instances where in the same pharmacy some simulated clients reported seeing a poster or leaflets whilst others did not. This may reflect human error, in that there could have been posters up that some simulated clients simply did not see because they were distracted by the busy pharmacy environment. Another explanation could be that since the simulated clients visited at different times, the materials were displayed on some days but not on others.

Strengths and weaknesses in the context of other studies

Previous studies using simulated patients to assess community pharmacy staff performance have focused on provision of over the counter medication [29, 32; 33] whereas our research examines the expanded role of pharmacy staff as agents for health behaviour change. However, we used similar methods: covert visits, blinding to time and number of visits to minimise detection [29, 32]. A key strength of our approach was the use of simulated clients with lived experience of smoking or smoking related health conditions such as asthma. Moreover, feedback from staff in our study indicated no detection of our simulated visits whilst detections were reported in other studies [29, 33].

In this study we assessed the initial interaction between pharmacy worker and smoker which usually takes place over the counter in community pharmacies. However, other researchers have audio-recorded consultations with stop smoking advisors allowing detailed examination of their interactions with clients which take place in a dedicated /private consulting room [8]. Due to ethical considerations related to obtaining patient consent, it was difficult to audio-record naturalistic interactions with pharmacy users within the time limitations of this study. In the main trial, we aim to supplement data from simulated client visits with audiotaped follow-up consultations between stop smoking advisors and actual service users.

One study [30] with a similar aim and methodological approach to ours randomly assigned pharmacies to two different scenarios [38]. With our method, all pharmacies were assessed against all six scenarios thus examining a broader range of skills and allowing a more thorough evaluation of potential gaps in service delivery. In the main trial we will use a balanced design where each pharmacy is exposed to each scenario which will allow more rigorous comparison of the degree to which the intervention is implemented between different pharmacies.

Implications for clinical practice and policy

The methods that we outline here could potentially be adapted to evaluate the effects of any training programme intended to modify the clinical practice of pharmacy staff. Given that government policy in the UK is to expand the range of clinical services provided in pharmacies [39, 40] methods to evaluate the effects of training may be useful in refining interventions and developing new training programmes.

Unanswered questions and future research

For peer review only - http://bmjopen.bmj.com/site/about/guidelines.xhtml

The simulated clients noted adequate display of intervention materials in the pharmacies. However, certain materials were displayed more prominently than others. In the main trial, there is a need to evaluate carefully the use of intervention materials and to understand reasons why certain materials are given more prominence and used more often than others. A better understanding of these factors may lead to development of more effective intervention materials which are more likely to be available to potential users of the intervention.

Author Contributions

SJ led development of the final version of the intervention and designed the fidelity assessment. WYJ and SJ recruited sites, delivered the training, and collected qualitative and quantitative data for this second pilot study. SJ, VM and TY analysed the data. LS led development of the initial intervention for the first pilot study, and gave critical comments on the manuscript alongside WYJ, VM, RS, TY, ST, CG and SE. SJ and RW drafted the paper with input from all other authors. All authors have seen and approved the final manuscript.

Acknowledgments

Mr Darush Attar who is a pharmacist, behaviour change specialist and national public health trainer specialised in smoking cessation, co-facilitated the STOP Intervention training with SJ and WYJ. This work has been previously presented as a thematic poster at the European Respiratory Society Milan 2017 International Congress.

Funding

This is a repilot study of the NIHR-funded STOP programme on which RW is chief investigator and CG, SE and ST are co-investigators. NIHR Programme grant RP-PG-0609-10181.

Competing interests None declared.

Ethics approval Ethical approval for the study was obtained from the Queen Mary Research Ethics Committee [Reference: QMREC1830a]

Provenance and peer review Not commissioned; externally peer reviewed.

Data sharing statement Study data (with identifying information removed) will be available, following completion of the project, on request from the study guarantor, Robert Walton, r.walton@qmul.ac.uk.

REFERENCES

1. Lancaster T, Stead LF: Individual behavioural counselling for smoking cessation. *Cochrane Database Syst Rev* 2005:CD001292.

2. Stead LF, Lancaster T: Group behaviour therapy programmes for smoking cessation. *Cochrane Database Syst Rev* 2005:CD001007.

3. Stead L, Perera R, Lancaster T: Telephone counseling for smoking cessation. *Cochrane Database Syst Rev* 2006, 2005, CD002850.

For peer review only - http://bmjopen.bmj.com/site/about/guidelines.xhtml

4. Michie S, Churchill S, West R: Identifying evidence-based competences required to deliver behavioural support for smoking cessation. *Ann Behav Med* 2011, 41(1):59–70.

5. West R, Stapleton J: Clinical and public health significance of treatments to aid smoking cessation. *Eur Respir Rev* 2008, 17(110):199–204.

6. Lorencatto F, West R, Michie S: Identifying evidence-based behaviour change techniques for smoking cessation in pregnancy. *Nicotine & Tobacco Research* 2012, 14(9):1019–1026. doi:10.1093/ntr/ntr324.

7. Raw M, Regan S, Rigotti NA, McNeill A: A survey of tobacco dependence treatment services in 36 countries. *Addiction* 2009, 104(2):279–87.

8. Lorencatto F, West R, Christopherson C, Michie S. Assessing fidelity of delivery of smoking cessation behavioural support in practice. *Implementation Science*. 2013; 8(1):40.

9. Bauld L, Bell K, McCullough L, Richardson L, Greaves L. The effectiveness of NHS smoking cessation services: a systematic review. Journal of Public Health. 2010 Mar 1;32(1):71-82.

10. Brose LS, West R, Michie S, Kenyon JA, McEwen A. Effectiveness of an online knowledge training and assessment program for stop smoking practitioners. Nicotine & Tobacco Research. 2012 Jan 16;14(7):794-800

11. Anderson C, Blenkinsopp A & Armstrong M, 2009. The contribution of community pharmacy to improving the public's health: summary report of the literature review 1990–2007. Five earlier reports at PharmacyHealthLink.

12. Latif A. & Boardman H. Community pharmacists' attitudes towards medicines use reviews and factors affecting the numbers performed. *Pharm World Sci*, 2008; 30:536–543

13. Saramunee K, Krska J, Mackridge A, Richards J, Suttajit S, Phillips-Howard P. How to enhance public health service utilization in community pharmacy?: General public and health providers' perspectives. Research in Social and Administrative Pharmacy. 2014;10(2):272-84.

14. Eades CE, Ferguson JS, O'Carroll RE. Public health in community pharmacy: a systematic review of pharmacist and consumer views. BMC public health. 2011;11(1):582.

15. Hudmon KS, Prokhorov AV, Corelli RL. Tobacco cessation counseling: pharmacists' opinions and practices. Patient education and counseling. 2006 Apr 30;61(1):152-60.

For peer review only - http://bmjopen.bmj.com/site/about/guidelines.xhtml

16. Sohanpal R, Rivas C, Steed L, MacNeill V, Kuan V, Edwards E, Griffiths C, Eldridge S, Taylor S, Walton R. Understanding recruitment and retention in the NHS community pharmacy stop smoking service: perceptions of smoking cessation advisers. BMJ open. 2016 Jul 1;6(7):e010921.

17. Sinclair H, Bond C, Lennox, AS, et al. An evaluation of a training workshop for pharmacists based on the Stages of Change model of smoking cessation. *Health Education Journal* 1997;56:296-312.

18. Sinclair HK, Bond CM, Lennox AS, et al. Training pharmacists and pharmacy assistants in the stage-of-change model of smoking cessation: a randomised controlled trial in Scotland. *Tob Control* 1998;7(3):253-61.

19. Maguire TA, McElnay JC, Drummond A. A randomized controlled trial of a smoking cessation intervention based in community pharmacies. *Addiction* 2001;96(2):325-31. doi:
10.1080/09652140020021062

20. Greenhalgh T, Macfarlane F, Steed L, Walton R. What works for whom in pharmacist led smoking cessation support: realist review. BMC Medicine 2016 14:209. DOI: 10.1186/s12916-016-0749-5

21. Rivas C, Sohanpal R, McNeill V,Steed L, Edwards E, Griffiths C, Eldridge S, Taylor SJC, Walton R. Defining effective communication strategies in the NHS community pharmacy stop smoking programme: a naturalistic, matched-pairs analysis of smoker consultations. BMJ Open 2017;7:e015664. doi: 10.1136/bmjopen-2016-015664

22. Bock BC, Hudmon KS, Christian J, Graham AL, Bock FR. A tailored intervention to support pharmacy-based counseling for smoking cessation. Nicotine & tobacco research. 2010 Jan 25;12(3):217-25.

23. Madurasinghe VW, Sohanpal R, James W-Y, Steed L, Eldridge SE, Taylor SJC, Griffiths CJ, Walton R. Smoking treatment optimisation in pharmacies (STOP): a cluster randomised pilot trial of a training intervention. Pilot and Feasibility Studies 2017:3:1 DOI: 10.1186/s40814-016-0120-9

24. Steed L, Sohanpal R, James WY, Rivas C, Jumbe S, Chater A, Todd A, Edwards E, MacNeill V, Macfarlane F, Greenhalgh T. Equipping community pharmacy workers as agents for health behaviour change: developing and testing a theory-based intervention. BMJ Open. 2017 Mar 22.

25. West R, Walia A, Hyder N, et al. Behavior change techniques used by the English Stop Smoking Services and their associations with short-term quit outcomes. *Nicotine Tob Res*
2010;12(7):742-7. doi: 10.1093/ntr/ntq074

26. Breitenstein SM, Gross D, Garvey CA, Hill C, Fogg L, Resnick B. Implementation fidelity in community-based interventions. Research in nursing & health. 2010 Apr 1;33(2):164-73.

For peer review only - http://bmjopen.bmj.com/site/about/guidelines.xhtml

27. Boutron I, Moher D, Altman D, Scultz K, Ravaud P: Extending the CONSORT statement to randomized trials of Non-pharmacologic treatment: explanation and elaboration. Annals of Internal Medicine 2008, 148:295–309

28. Rixon L, Baron J, McGale N, Lorencatto F, Francis J, Davies A. Methods used to address fidelity of receipt in health intervention research: a citation analysis and systematic review. BMC health services research. 2016 Dec;16(1):663.

29. Watson MC, Skelton JR, Bond CM, Croft P, Wiskin CM, Grimshaw JM, Mollison J. Simulated patients in the community pharmacy setting–Using simulated patients to measure practice in the community pharmacy setting. Pharmacy World and Science. 2004 Feb 1;26(1):32-7.

30. Saba M, Diep J, Bittoun R, Saini B. Provision of smoking cessation services in Australian community pharmacies: a simulated patient study. International journal of clinical pharmacy. 2014 Jun 1;36(3):604-14.

31. Watson MC, Norris P, Granas AG. A systematic review of the use of simulated patients and pharmacy practice research. International Journal of Pharmacy Practice. 2006 Jun 1;14(2):83-93.

32. Watson MC, Cleland JA, Bond CM. Simulated patient visits with immediate feedback to improve the supply of over-the-counter medicines: a feasibility study. Family practice. 2009 Oct 14;26(6):532-42.

33. Weiss MC, Booth A, Jones B, Ramjeet S, Wong E. Use of simulated patients to assess the clinical and communication skills of community pharmacists. Pharmacy world & science. 2010 Jun 1;32(3):353-61.

34. Robson J, Dostal I, Madurasinghe V, Sheikh A, Hull S, Boomla K, Page H, Griffiths C, Eldridge S. The NHS Health Check programme: implementation in east London 2009–2011. BMJ open. 2015 Apr 1;5(4):e007578.

35. Snow J, Mann M. Qualtrics Survey Software: Handbook for Research Professional. Provo, UT: Qualtrics Labs.

36. Rosvall A, Carlson E. Registered nurses' perception of self-efficacy and competence in smoking cessation after participating in a web-based learning activity. Journal of clinical nursing. 2017 Dec;26(23-24):4777-85.

37. Williams DM, Newsom JF, Brock TP. An evaluation of smoking cessation-related activities by pharmacists. Journal of the American Pharmaceutical Association (1996). 2000 Mar 1;40(3):366-70.

38. Brose LS, West R, Michie S, et al. Evaluation of Face-to-Face Courses in Behavioural Support for Stop Smoking Practitioners. Journal of Smoking Cessation 2012;7:25-30. doi:doi:10.1017/jsc.2012.6

For peer review only - http://bmjopen.bmj.com/site/about/guidelines.xhtml

39. Donovan GR, Paudyal V. England's Healthy Living Pharmacy (HLP) initiative: Facilitating the engagement of pharmacy support staff in public health. Research in social and administrative pharmacy. 2016 Mar 1;12(2):281-92.

40. Twigg MJ, Wright D, Kirkdale CL, Desborough JA, Thornley T. The UK Pharmacy Care Plan service: Description, recruitment and initial views on a new community pharmacy intervention. PloS one. 2017 Apr 3;12(4):e0174500.

For peer review only

Tables

Table 1: Detailed description of the STOP Intervention

For peer review only

PHARMACY SITE INITIATION VISIT	Content	Theoretical Basis [a]	Behaviour change techniques [b]
	Explain the study to pharmacist in charge or manager.<br>Mention potential revenue stream from smoking cessation.<br><br>Emphasise to staff how this fits well with their wider role in health promotion.<br><br>Raise awareness in all staff in preparation for invitation to training.<br><br>Communicate the advantages of the STOP intervention over usual practice, i.e. it is brief and show how it fits with overall 'pharmacy' identity.<br><br>Address pre implementation concerns.<br><br>Provide financial incentive for attending training (only received on completion of training)<br><br>Emphasise backing from local and national opinion leaders and organisations (e.g. Local Pharmaceutical Committee, Royal Pharmaceutical Society, local CCGs and public health commissioners	Adoption by individuals: concerns in preadoption stage (DIT)<br><br>The innovation: compatibility;<br>relative advantage; low complexity (DIT)<br><br>Outer context: incentives (DIT)<br><br>Diffusion and dissemination: opinion leaders (DIT)	10.2 Material reward (behaviour)<br><br>9.1 Credible source<br><br>1.2 Problem Solving<br><br>6.3 Information about others' approval
**TRAINING SESSION**	**Content**	**Theoretical Basis**	**Behaviour change techniques**
**Introduction**	General orientation to STOP programme and aims of training.<br><br>Emphasise backing from local and national opinion leaders and organisations (e.g. Local Pharmaceutical Committee, Royal Pharmaceutical Society, local CCGs and public health commissioners<br><br>Discussion of impact of advisor behaviour on client stop smoking outcomes so far and health benefits to patients from stopping smoking.	Outcome Expectancies (SCT)<br><br>Diffusion and dissemination: opinion leaders (DIT)	5.1 Information on health consequences of behaviour<br><br>9.1 Credible source<br><br>10.6 Nonspecific incentive<br><br>15.1 Verbal persuasion about capability

For peer review only

	Delivered in mixed groups of pharmacists and other pharmacy workers to promote cohesive working practices within the individual pharmacies.	Implementation and routinisation: organizational structure (DIT)	
**Why are we here?**	Smoking facts and exploration of motivation for helping smokers to quit with feedback.<br><br>Discuss focus on pharmacy setting, emphasising the non-medication related, professional and public health aspects of the pharmacy role.<br><br>Does engaging and supporting smokers' quit fit with role identity, any barriers? Encourage self-perception as supporters and providers of health, how one will feel if help smokers quit.<br><br>Emphasise the non-medication related, professional and public health aspects of the pharmacy role, promote a person-centred rather than product-centred ethos and foster a strong sense of professionalism	Intrinsic and extrinsic motivators (SDT)<br><br>The innovation: compatibility (DIT)	5.6 Information about emotional consequences<br><br>9.2 Pros and cons<br><br>6.3 Information about others approval<br><br>13.1 Identification of self as a role model<br><br>15.3 Focus on past success
**Engaging Clients**	Celebrate successful cases.<br><br>Group exercise and discussion on difficult and easy clients to engage – potential problems and solutions.<br><br>Addressing pharmacy workers beliefs and attitudes e.g. prejudgement of success or failure.	Self-efficacy (SCT)<br>Modelling (SCT)<br>Vicarious learning (SCT)	1.2 Problem solving,<br>8.1 Behavioural practice and rehearsal<br>9.2 Pros and cons<br>13.3 Incompatible beliefs<br>15.3 Focus on past success
**Patient-centred approach: Building rapport & shift of focus**	Introduction of patient centred approach using group exercise. Group identification and discussion of the importance of utilising basic communication skills (rapport, active listening, questioning).<br><br>Review how patient-centred approach can be incorporated into smoking cessation interactions for better patient outcomes<br><br>Role-play demonstration with senior pharmacist, participant practice. How to maximise opportunity with environmental resources e.g. staff wearing STOP badges to prompt client interaction, STOP posters<br><br>Emphasise predictable improved results, simplicity of use and	Self-efficacy (SCT)<br>Modelling (SCT)<br>Vicarious learning (SCT)<br>The innovation: relative advantage; compatibility; low complexity (DIT)	4.1 Instruction on performance of behaviour,<br>6.1 Demonstration of behaviour<br>7.1 Prompts and cues<br>8.1 Behavioural practice and rehearsal<br>8.2 Behaviour substitution<br>8.6 Generalisation of target behaviour<br>9.2 Pros and cons<br>9.3 Comparative imagining of future outcomes

For peer review only - http://bmjopen.bmj.com/site/about/guidelines.xhtml

	benefits over usual practice.		
**NCSCT Knowledge Review**	Review group's NCSCT knowledge with a quiz and general feedback	Self-efficacy (SCT)	1.6 Discrepancy between current behaviour and goal<br>9.1 Credible source
**Pharmacy role in smoking cessation**	Discuss individual pharmacies' NHS Stop Smoking service structure and purpose of smoking treatment, with experienced advisers sharing current and best practice. Group reflection on challenges	Homophily (DIT)	1.7 Review outcome goal(s)
**Behaviour change as smoking cessation treatment**	Emphasise behaviour change support as part of smoking cessation treatment within NHS SSS.<br><br>Information on how to assess someone's readiness to quit smoking using 1-10 scales.	Self-efficacy (SCT)<br>Self-regulation (SCT)<br>The innovation: compatibility (DIT)	4.1 Instruction how to perform behaviour<br>8.1 Behavioural practice and rehearsal
**Behaviour change using patient centred approach in Smoking Cessation Treatment: Double Whammy**	Brainstorm factors that influence behaviour change – (role of beliefs, capability, opportunity alongside knowledge)<br><br>How to elicit individuals' motivations, barriers and potential strategies to change behaviour versus offering solutions. Using 'What else questions'.<br><br>Understanding the 'non-smoker identity' and how to communicate to client<br><br>Demonstration & Role play<br><br>What makes this client centred approach difficult - advantages, disadvantages, barriers and strategies to aid implementation	Outcome Expectancies (SCT)<br>Modelling (SCT)<br>Self-efficacy (SCT)<br>The innovation: fuzzy boundaries (DIT)	1.2 Problem Solving<br>4.1 Instruction on how to perform behaviour<br>6.1 Demonstration of behaviour<br>8.1 Behavioural practice and rehearsal<br>9.2 Pros and Cons
**Client engagement in pharmacy settings: Planning a Quit & Dealing with Lapses**	Discuss planning a quit and how to help people make a specific plan using a SMART approach. Go over ways to discuss with lapses and provide supportive praise.<br><br>Discussion of how to talk about willpower and the role of the open door.<br><br>Watch and reflect on video of strong and weak consultations of quit planning.	Modelling (SCT)<br><br>Self-efficacy (SCT)	1.1 Goal setting (behaviour)<br>1.2 Problem solving<br>3.1 Social support (unspecified)<br>4.1 Instruction on how to perform behaviour<br>6.1 Demonstration of behaviour<br>8.1 Behavioural practice and rehearsal

For peer review only - http://bmjopen.bmj.com/site/about/guidelines.xhtml

	Demonstration & Role play		
**Client engagement in pharmacy settings: Goal Setting & Making a Commitment**	Facilitate goal setting and elicit verbal commitment from participants.<br><br>Demonstration (via video)<br><br>Practice cohesive working amongst trainees through role play using multiple scenarios and observer feedback	Modelling (SCT)	1.1 Goal Setting (behaviour)<br>1.9 Commitment<br>3.1 Social support (unspecified)<br>8.1 Behavioural practice and rehearsal<br>15.1 Verbal persuasion about capability
**Implementing STOP**	Review how to implement STOP in practice (i.e. prompts and WhatsApp support) by facilitating discussion of implementation plans alongside facilitators or barriers with pharmacy team<br><br>Highlight use of local champions and prompts/cues including the Double Whammy (a desk calendar with visual cues and example questions to ask) to prompt client interaction.<br><br>Highlight ongoing social support via WhatsApp<br>Promote adaptation of non-core elements of the intervention through a prompted pharmacy team meeting to discuss implementation of the intervention according to the needs of each individual pharmacy e.g. appointment of individual champions, monthly 'STOP' smoking days	Self-regulation (SCT)<br>Intrinsic/Extrinsic Motivators (SDT)<br>The innovation: augmentation/support (DIT)<br>The innovation: trialability; reinvention; fuzzy boundaries; champions (DIT)<br><br>Implementation & routinisation: organisational structure (DIT)	4.1 Instruction on how to perform behaviour<br>1.2 Problem Solving<br>7.1 Prompts and Cues<br>3.2 Social support (practical)<br>1.4 Action planning<br><br>10.1 Material incentive (behaviour)
**End of Session**	Participants provided with a certificate for attending the training linked to CPD (endorsed by RPS)<br><br>Provide financial reward for those who have completed intervention training	Outer context: incentives (DIT)	10.2 Material reward
**6 WEEK BOOSTER VISIT**	**Content**	**Theoretical Basis**	**Behaviour change techniques**
**Feedback on smoker engagement and update on STOP implementation**	Identification of organizational barriers, facilitators to implementing STOP in individual pharmacies. Facilitating action plans to implement STOP in their pharmacy.<br><br>Any further thoughts on how the intervention can be adapted to local circumstances?<br><br>Review pharmacy staffs' self-efficacy of skills	The innovation: fuzzy boundaries (DIT)<br>Adoption by individuals: concerns in preadoption stage (DIT)<br><br>The innovation: reinvention	1.1 Goal Setting<br>1.2 Problem solving<br>1.4 Action Planning<br>1.5 Review behaviour goal(s)<br>1.6 Discrepancy between current behaviour and goal<br>1.7 Review outcome goal<br>1.9 Commitment

For peer review only - http://bmjopen.bmj.com/site/about/guidelines.xhtml

	Troubleshoot based on performance feedback, assessment by simulated clients* and staff's self-reported self-efficacy<br><br>*Simulated clients are trained actors who approach staff in intervention pharmacies using smoking-related scenarios to assess client engagement and evaluate presence of STOP smoking environmental cues (posters, badges)	The innovation: augmentation/support (DIT)<br><br>Self-efficacy (SCT)<br><br>The innovation: trialability (DIT)	2.2 Feedback on behaviour<br>2.7 Feedback on outcome of behaviour<br><br>7.1 Prompts and cues<br><br>8.3 Habit formation

Footnote
[a] DIT refers to 'Diffusion of Innovation' Theory. See Greenhalgh T, et al. Diffusion of innovations in service organizations: systematic review and recommendations. The Milbank Quarterly. 2004 Dec;82(4):581-629.
SCT refers to 'Social Cognitive Theory'. See Bandura A. Health promotion from the perspective of social cognitive theory. Psychology and health. 1998 Jul 1;13(4):623-49.
[b] derived from the Behaviour Change taxonomy (Michie S. et al The behavior change technique taxonomy (v1) of 93 hierarchically clustered techniques: building an international consensus for the reporting of behavior change interventions. Annals of behavioral medicine. 2013 Mar 20;46(1):81-95.

For peer review only

Table 2: Pharmacy Staff Demographics

CHARACTERISTICS		SUPPORT STAFF (n=16)	STOP SMOKING ADVISORS (n=4)	Total
Mean age in years (Range)		29 (16-49)	37 (30-54)	30 (16-54)
Male (%)		38	100	50
Graduate or higher (%)		25	100	40
Never smoked (%)		75	100	80
Job titles	**Counter Assistant**	10		
	**Dispensing Chemist**	4		
	**Trainee pharmacist**	2		
	**Pharmacist**		2	
	**Pharmacist technician**		1	
	**Business Manager**		1	

Table 3: Simulated clients demographics

ID	Age	Gender	Ethnicity	Education	Smoking Status
01	49	Male	White	Postgraduate	Never smoked
02	56	Female	White British	Other	Ex-smoker
03	54	Male	Mixed	Graduate	Ex-smoker
04	32	Male	Black	Graduate	Never smoked
05	22	Female	Mixed	NVQ L3	Ex-smoker
06	58	Female	White British	Professional (CPCAB)	Ex-smoker

For peer review only

Table 4: Client engagement ratings from simulated smokers

PHARMACY SITE	% of pharmacy staff trained	*Client Engagement Ratings by simulated clients*						
		**1**	**2**	**3**	**4**	**5**	**6**	**Average score**
**S06**	**100**	36	34	36	34	34	29	35
**S02**	**60**	29	14	18	29	6	14	18
**S01**	**57**	18	14	16	25	27	6	18
**S05**	**40**	10	4	20	15	28	14	15
**S03**	**33**	14	24	25	27	22	20	22

LEGEND
- ☐ Completed STOP training
- ■ Did not attend training
- ▒ Unable to identify

Table 5: Mean Client Engagement Scores for trained *vs* untrained staff

	ATTENDED STOP TRAINING	N	Mean	Std. Dev.
OVERALL CLIENT ENGAGEMENT	No	12	16.9	7.7
	Yes	18	24.4	9.0
BUILDING RAPPORT	No	12	6.7	3.7
	Yes	18	9.1	2.3
CONVERSATION	No	12	10.3	5.1
	Yes	18	15.3	7.1

For peer review only

Table 6: Average simulated client fidelity assessment ratings including display of smoking cessation materials and communication skills

	SC 1	SC 2	SC 3	SC 4	SC 5	SC 6
**Display of Smoking Cessation Materials**						
NHS SSS Poster / audio information	Y= 3	Y= 1	Y= 0	Y= 5	Y= 0	Y= 2
NHS SSS Leaflets	Y= 4	Y= 5	Y= 3	Y= 5	Y= 5	Y= 5
STOP study poster	Y= 1	Y= 1	Y= 2	Y= 3	Y= 0	Y= 3
STOP study badge	Y= 0	Y= 1	Y= 1	Y= 1	Y= 0	Y= 1
***Rapport with clients**						
Good use of body language	2	2	2	3	3	2
Good listening skills	2	2	2	3	3	2
Use of open questions	1	1	2	1	2	2
Picking up client's verbal or visual cues	2	2	2	2	3	2
***Conversation**						
Initiate conversation on smoking in response to cues	2	1	2	3	2	2
Raise smoking directly	2	1	1	2	2	0
Raise smoking indirectly	1	0	1	1	0	0
Tell client about available SSS in pharmacy	2	3	3	3	2	2
Highlight free or subsidised service	2	3	3	2	2	1
Highlight facts on SSS high quit rates	1	1	1	1	1	1
Ask client about service referral	2	1	3	2	2	1
Close conversation with 'come back anytime' for help	2	2	2	3	2	2

Key
SC = simulated client
'Y=' refers to the total number of times a simulated client ticked to confirm display of a smoking cessation material from their 5 pharmacy visits
* Numbers here refer to average client engagement scores assigned by simulated smokers across their 5 pharmacy visits. Range of 0-3, where 0 indicates no rapport or conversation and 3 indicates very good rapport or conversation

For peer review only

Figures

Figure 1: STOP Programme Final Logic Model

Figure 2: Smoking-related scenarios

Figure 3: Fidelity Assessment Questionnaire

For peer review only

For peer review only - http://bmjopen.bmj.com/site/about/guidelines.xhtml

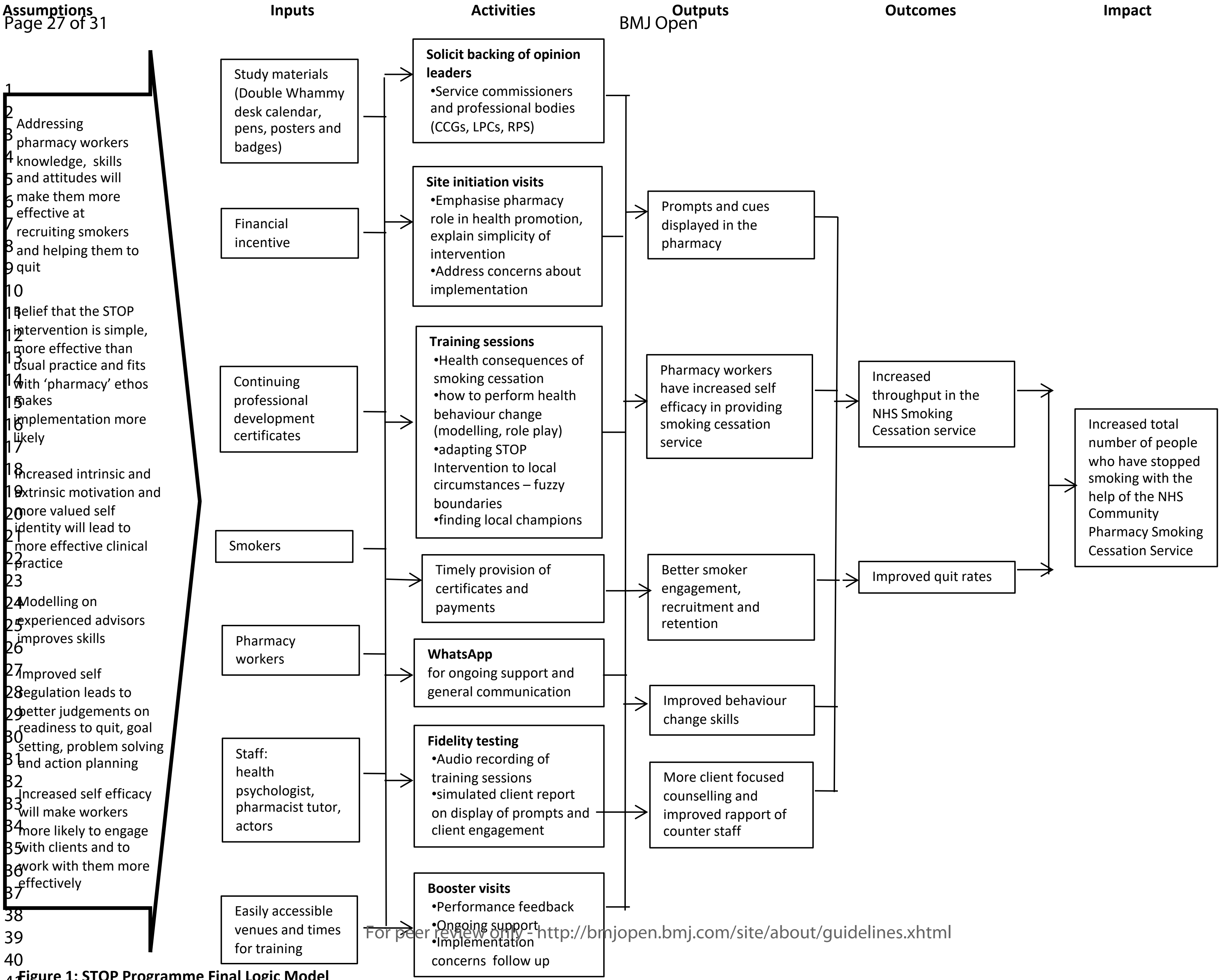

For peer review only - http://bmjopen.bmj.com/site/about/guidelines.xhtml

Figure 1: STOP Programme Final Logic Model

Figure 2: Brief scenario outlines

**Scenario 1**<br>Individual picks up SSS visual resource and reads through, attends counter and asks if the pharmacy offers advice on how to stop smoking. Has discovered teenage child smokes and wants them to stop.<br><br>Actor required: middle aged woman, white	**Scenario 2**<br>Individual attends counter coughing, with cigarettes obviously on display e.g. holding in hand and putting on counter. Individual tells pharmacy staff they have asthma and need advice on how to stop smoking.<br><br>Actor required: middle aged male, white	**Scenario 3**<br>Individual attends counter with non-smoking related request e.g. purchase pregnancy vitamins. Has cigarettes visible and obviously on display e.g. holding in hand and putting on counter.<br><br>Actor required: middle aged male, ethnic minority
**Scenario 4**<br>Individual attends counter wheezing, with asthma pump e.g. holding in hand and putting on counter. Individual tells pharmacy staff they have asthma and COPD but still smoke. Asthma is getting worse so they need advice on how to stop smoking.<br><br>Actor required: middle aged female, white	**Scenario 5**<br>Individual attends the pharmacy counter, complaining of a sore throat and need of lozenges. Explains that they went partying last night with friends and was smoking so maybe sore throat is linked to that. Friend and mother nagging that sore throat is due to smoking. Ask pharmacy worker what they think it could be?<br><br>Actor required: young female, ethnic minority	**Scenario 6**<br>Individual attends counter, complaining of stress from girlfriend/mum to stop smoking because he has been getting sore throats a lot lately. Not sure if he wants to quit but thought he should check out what the pharmacy have to offer. Saw a sign that says pharmacy has a Stop Smoking Service.<br><br>Actor required: young male, ethnic minority

For peer review only

STOP

Actor ID: ___ ___

Fidelity Assessment Questionnaire

Date: DD / MM / YEAR	Time: __ : __ hours	Scenario number:	Pharmacy ID

Environment

Were the following visible within the pharmacy:-	***Tick if seen***
NHS Stop Smoking Service poster or audiovisual information	
NHS Stop Smoking Service leaflets	
STOP Study Poster	
Staff wearing STOP Study Badges	

Rapport

To what extent did the pharmacy worker demonstrate:- (circle a number)

a) good body language e.g. eye contact, attentiveness 0 1 2 3
b) good listening skills e.g. focused on conversation 0 1 2 3
c) use of open questions 0 1 2 3
d) picking up on client's verbal or visual cues 0 1 2 3

Conversation

To what extent did the pharmacy worker:- (circle a number)

a) Initiate conversation of smoking in response to a verbal or visual prompt 0 1 2 3
b) Raise smoking directly e.g. do you smoke 0 1 2 3
c) Raise smoking indirectly e.g. by talking about second hand smoke 0 1 2 3
d) Tell you there is a smoking cessation service available in the pharmacy 0 1 2 3
e) Highlight that the service is free aside from NRT if pay for prescriptions 0 1 2 3
f) Highlight that people who use the service are 4 times more likely to quit 0 1 2 3
h) Ask you whether you want a referral to the service 0 1 2 3
i) Close conversation by saying you can come back anytime for help/support 0 1 2 3

Additional comments:	Total score:

For peer review only

STROBE Statement—Checklist of items that should be included in reports of ***cross-sectional studies***

	Item No	Recommendation	Page no.
**Title and abstract**	1	(*a*) Indicate the study's design with a commonly used term in the title or the abstract	1, 2
		(*b*) Provide in the abstract an informative and balanced summary of what was done and what was found	2
**Introduction**			
Background/rationale	2	Explain the scientific background and rationale for the investigation being reported	3,4
Objectives	3	State specific objectives, including any prespecified hypotheses	4
**Methods**			
Study design	4	Present key elements of study design early in the paper	4
Setting	5	Describe the setting, locations, and relevant dates, including periods of recruitment, exposure, follow-up, and data collection	4
Participants	6	(*a*) Give the eligibility criteria, and the sources and methods of selection of participants	4, 5
Variables	7	Clearly define all outcomes, exposures, predictors, potential confounders, and effect modifiers. Give diagnostic criteria, if applicable	6
Data sources/ measurement	8*	For each variable of interest, give sources of data and details of methods of assessment (measurement). Describe comparability of assessment methods if there is more than one group	*6*
Bias	9	Describe any efforts to address potential sources of bias	6
Study size	10	Explain how the study size was arrived at	6
Quantitative variables	11	Explain how quantitative variables were handled in the analyses. If applicable, describe which groupings were chosen and why	6
Statistical methods	12	(*a*) Describe all statistical methods, including those used to control for confounding	6
		(*b*) Describe any methods used to examine subgroups and interactions	
		(*c*) Explain how missing data were addressed	
		(*d*) If applicable, describe analytical methods taking account of sampling strategy	
		(*e*) Describe any sensitivity analyses	
**Results**			
Participants	13*	(a) Report numbers of individuals at each stage of study—eg numbers potentially eligible, examined for eligibility, confirmed eligible, included in the study, completing follow-up, and analysed	6
		(b) Give reasons for non-participation at each stage	
		(c) Consider use of a flow diagram	
Descriptive data	14*	(a) Give characteristics of study participants (eg demographic, clinical, social) and information on exposures and potential confounders	6
		(b) Indicate number of participants with missing data for each variable of interest	
Outcome data	15*	Report numbers of outcome events or summary measures	7
Main results	16	(*a*) Give unadjusted estimates and, if applicable, confounder-adjusted estimates and their precision (eg, 95% confidence interval). Make clear which confounders were adjusted for and why they were included	6,7

For peer review only

		(*b*) Report category boundaries when continuous variables were categorized	6,7
		(*c*) If relevant, consider translating estimates of relative risk into absolute risk for a meaningful time period	
Other analyses	17	Report other analyses done—eg analyses of subgroups and interactions, and sensitivity analyses	
**Discussion**			
Key results	18	Summarise key results with reference to study objectives	8, 9
Limitations	19	Discuss limitations of the study, taking into account sources of potential bias or imprecision. Discuss both direction and magnitude of any potential bias	9
Interpretation	20	Give a cautious overall interpretation of results considering objectives, limitations, multiplicity of analyses, results from similar studies, and other relevant evidence	10
Generalisability	21	Discuss the generalisability (external validity) of the study results	10
**Other information**			
Funding	22	Give the source of funding and the role of the funders for the present study and, if applicable, for the original study on which the present article is based	11

*Give information separately for exposed and unexposed groups.

Note: An Explanation and Elaboration article discusses each checklist item and gives methodological background and published examples of transparent reporting. The STROBE checklist is best used in conjunction with this article (freely available on the Web sites of PLoS Medicine at http://www.plosmedicine.org/, Annals of Internal Medicine at http://www.annals.org/, and Epidemiology at http://www.epidem.com/). Information on the STROBE Initiative is available at www.strobe-statement.org.

For peer review only
